# Analysis of DNAs associated with coconut foliar decay disease implicates a unique single-stranded DNA virus representing a new taxon

**DOI:** 10.1038/s41598-018-23739-y

**Published:** 2018-04-09

**Authors:** Bruno Gronenborn, John W. Randles, Dennis Knierim, Quentin Barrière, H. Josef Vetten, Norman Warthmann, David Cornu, Tiata Sileye, Stephan Winter, Tatiana Timchenko

**Affiliations:** 1 Institute for Integrative Biology of the Cell, UMR9198, CNRS, Université Paris-Sud, CEA, Avenue de la Terrasse, 91198 Gif sur Yvette, France; 2 School of Agriculture Food and Wine, University of Adelaide, Glen Osmond, South Australia 5064 Australia; 3 Deutsche Sammlung von Mikroorganismen und Zellkulturen GmbH (DSMZ) Messeweg 11/12, 38104 Braunschweig, Germany; 4 Im Spargelfeld 1, 38162, Cremlingen, Germany; 5 Research School of Biology, The Australian National University, Linnaeus Way, Canberra, ACT 2601 Australia; 6 Vanuatu Agricultural Research and Technical Centre, Santo, Vanuatu

## Abstract

The unique ecology, pathology and undefined taxonomy of coconut foliar decay virus (CFDV), found associated with coconut foliar decay disease (CFD) in 1986, prompted analyses of old virus samples by modern methods. Rolling circle amplification and deep sequencing applied to nucleic acid extracts from virion preparations and CFD-affected palms identified twelve distinct circular DNAs, eleven of which had a size of about 1.3 kb and one of 641 nt. Mass spectrometry-based protein identification proved that a 24 kDa protein encoded by two 1.3 kb DNAs is the virus capsid protein with highest sequence similarity to that of grabloviruses (family *Geminiviridae*), even though CFDV particles are not geminate. The nine other 1.3 kb DNAs represent alphasatellites coding for replication initiator proteins that differ clearly from those encoded by nanovirid DNA-R. The 641 nt DNA-gamma is unique and may encode a movement protein. Three DNAs, alphasatellite CFDAR, capsid protein encoding CFDV DNA-S.1 and DNA-gamma share sequence motifs near their replication origins and were consistently present in all samples analysed. These DNAs appear to be integral components of a possibly tripartite CFDV genome, different from those of any *Geminiviridae* or *Nanoviridae* family member, implicating CFDV as representative of a new genus and family.

## Introduction

Coconut foliar decay (CFD), a severe disease of coconut palms in Vanuatu, was first described around 1964^1,2^, and a single-stranded (ss) DNA was found in 1985 when initial attempts at studying the aetiology of the disease were made^3,4^. It was used at first as a molecular marker for disease diagnosis^2,5,6^, then for identifying 20 nm icosahedral virions of a new plant virus, coconut foliar decay virus (CFDV)^6^, and also for supporting a persistent-circulative mode of virus transmission by *Myndus taffini* (Hemiptera: Ciixidae)^7^. In an effort to analyse and sequence the virus genome a single ssDNA of 1291 nt containing an inverted repeat with the potential of forming a stem-loop structure and encoding several putative proteins was identified^8^. This structure and the fact that the major potentially encoded protein resembled the replication initiator (Rep) proteins of ssDNA elements multiplying by rolling circle replication (RCR)^9^ characterized it at the time as circo- or geminivirus-like^8^. While our knowledge of geminiviruses and RCR elements including ssDNA viruses of animals and plants has progressed tremendously since and the number of new ssDNA virus species, genera and families has substantially increased^10–15^, the properties of the single sequenced circular ssDNA of CFDV has left the virus as an unassigned species within the family *Nanoviridae*^16^. Moreover, its sequence (GenBank acc. no. M29963) now shows that it is related phylogenetically to the alphasatellites associated with geminiviruses and nanovirids, that is, members of either the *Babuvirus* or *Nanovirus* genus in the *Nanoviridae* family.

The possibility that additional CFD-associated DNAs exist was supported by the detection of two or three DNAs in extracts of diseased palms by non-denaturing polyacrylamide gel electrophoresis^3^. We therefore decided to apply rolling circle amplification (RCA)^17–19^ to DNA from both CFD-associated virions and nucleic acid preparations from CFD-affected coconut palms to obtain more information about the genome of this enigmatic virus. Here we show that although CFDV combines features of geminiviruses and nanovirids, its other features suggest that it represents a distinct new taxon of circular ssDNA plant viruses for which we propose the genus name *Cofodevirus* (**co**conut **fo**liar **de**cay **virus)** and a tentative family name *Naminiviridae* reflecting the combination of characteristics of both *Nanoviridae* and *Geminiviridae* family members in CFDV.

## Results

### Identification of CFDV DNAs

Virion preparations from symptomatic leaves of two diseased hybrid coconut palms (sample pool CFD3) collected in 1988 and two diseased ‘Malayan Red Dwarf’ (MRD) coconut palms (sample pool CFD9) collected in 1989 (Table S1) served for RCA. Treatment of the amplified DNA by restriction endonucleases *Aat*II, *Eco*RI, *Bam*HI, *Kpn*I, *Age*I and *Sal*I yielded linear DNAs of 1.3 kb, and using *Kpn*I an additional 0.7 kb DNA was observed upon electrophoresis. Sequence analysis of the restricted and cloned RCA products revealed a total of ten different circular DNAs, nine of them ranging in length between 1252 and 1291 nucleotides (nt) and one consisting of 641 nt (Table S2).

**Figure 1:**
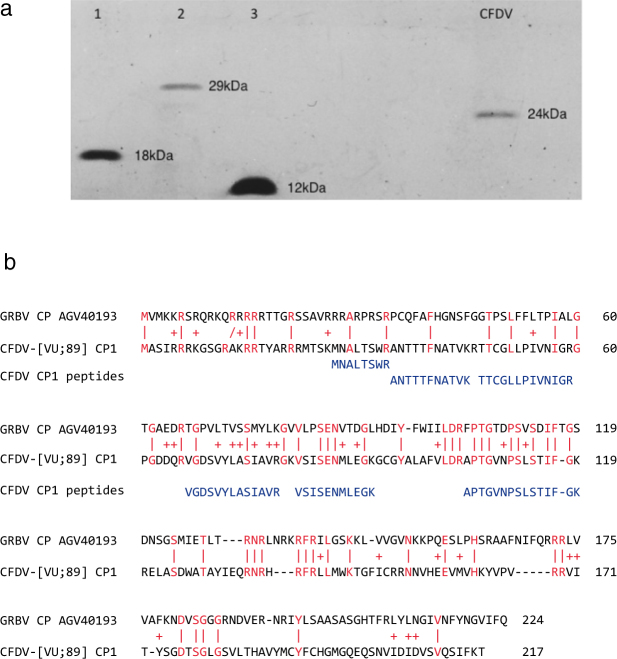
Identification of the CFDV capsid protein. (**a**) SDS-PAGE showing that CFDV has a capsid protein of Mr ~24 kDa as estimated using (lane 1) tobacco mosaic virus capsid protein (18 kDa), (lane 2) carbonic anhydrase (29 kDa) and (lane 3) cytochrome c (12 kDa) as size markers. (**b**) Comparison of CFDV CP1, encoded by CFDV [VU;89] DNA-S.1, with a capsid protein of a grablovirus, here GRBV protein AGV40193. Identical amino acids are displayed in red and marked by (|), similar amino acids are marked by (+). Tryptic peptides of CFDV particle-derived capsid protein, identified by mass spectrometry, are shown at matching positions below the protein sequence of CFDV CP1.

We found three types of DNA. DNA-S.1 and DNA-S.2 shared 90% sequence identity and carried open reading frames (ORFs) with a coding potential for proteins of respectively 217 and 215 amino acids (Table 1). The deduced proteins shared highest similarities (E-values of 2.4e-5 to 2e-4 in BlastP searches; 19–24% depending on alignment algorithms; see also Fig. 1b) with the capsid proteins (CP) of the grapevine-infecting grabloviruses^20,21^ as well as desmodium mottle virus (DesMoV)^22^. The deduced size (24 kDa) of the two respective CFDV capsid proteins is in good agreement with the relative molecular mass estimated by SDS-polyacrylamide gel electrophoresis (PAGE) for the protein obtained from purified CFDV particles (Fig. 1a). The protein band at 24 kDa prepared from virion-derived capsid protein (only from DNA-S.1 containing samples) was identified unambiguously by liquid chromatography-tandem mass spectrometry (LC-MS/MS) as CFDV CP1 (six trypsin-generated peptides with significant ion scores) (Fig. 1b). We did not find any capsid-derived peptides phosphorylated at serines, threonines or tyrosines. Following the nomenclature of nanovirid genome components that encode their respective capsid proteins on individual DNAs^16^ we named the CFDV CP-encoding genome components DNA-S.1 and DNA-S.2.

**Table 1:** CFD-associated DNAs: Summary of relevant features.

DNA	Accession no.	Length, nt.	ORF1 amino acids^a^	ORF1 protein function	ORF2	ORF3	ORF4
CFDV-[VU;89] DNA-S.1	MF926436	1286	217 (+)	CP	117 (+)		
CFDV-[VU;89] DNA-S.2	MF926439	1263	215 (+)	CP			
CFDV-[VU;89] DNA-gamma	MF926441	641	95 (+)	MP?	109 (−)	99 (−)	
CFDAR-[VU;89]	MF926434	1271	290 (+)	Rep	145 (+)		
CFDA1-[VU;89]	MF926424	1291	290 (+)	Rep	153 (+)		
CFDA2-[VU;89]	MF926426	1277	289 (+)	Rep	145 (+)	165 (−)	101 (−)
CFDA3-[VU;89]	MF926427	1252	287 (+)	Rep	85 (+)		
CFDA4-[VU;89]	MF926429	1276	290 (+)	Rep	152 (+)	139 (+)	248 (−)
CFDA5-[VU;89]^b^	MF926430	1295	290 (+)	Rep	125 (+)	207 (−)	100 (−)
CFDA6-[VU;88]	MF926431	1264	287 (+)	Rep			
CFDA7-[VU;89]	MF926432	1259	290 (+)	Rep	108 (−)		
CFDA8-[VU;15]^b^	MF926433	1271	290 (+)	Rep	152 (+)	172 (−)	

^a^Number of deduced amino acids, ORF orientation, (+) or (−) sense, and possible functions of deduced respective proteins are indicated: capsid protein (CP), potential movement protein (MP?) and replication initiator protein (Rep). Functions of potential proteins encoded by ORFs 2, -3, -4 are unknown.

^b^CFDA5 and CFDA8 were first discovered by deep sequencing and subsequently confirmed by PCR and cloning.

**Figure 2:**
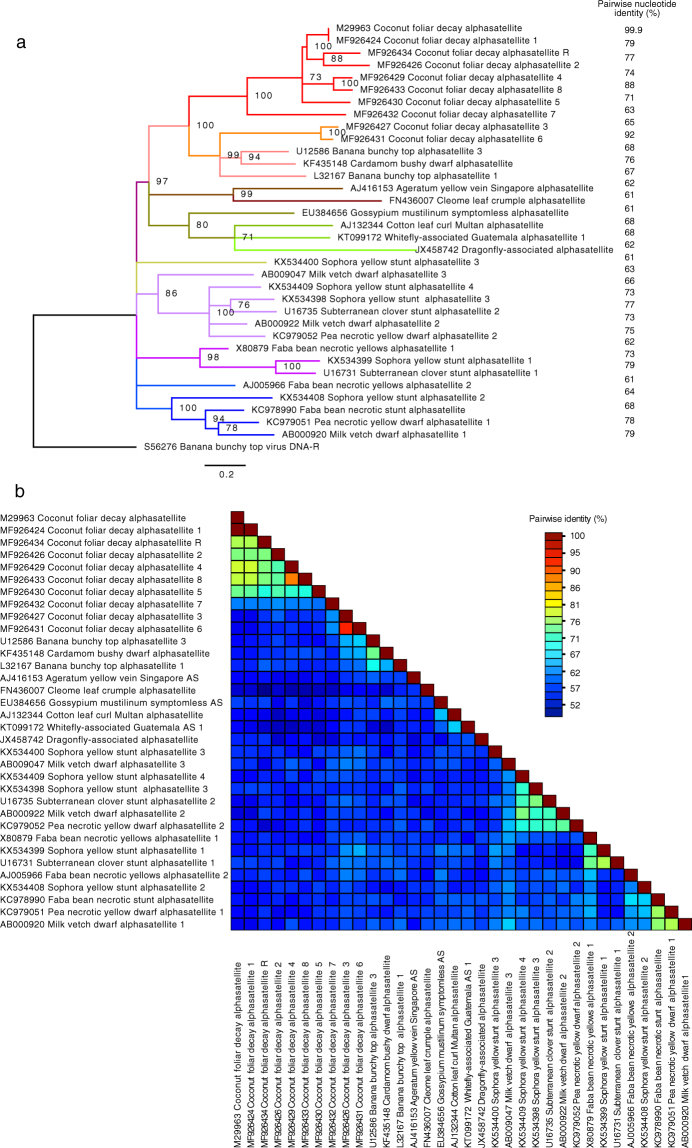
Maximum likelihood phylogenetic tree of selected alphasatellite DNAs and pairwise sequence identity plot. Apart from all coconut foliar decay alphasatellites only representatives of the most distantly related alphasatellites were chosen for comparison^25^. (**a**) The PhyML^63^ tree was rooted using the BBTV DNA-R sequence (S56276). Branch support (% bootstrap) is indicated. Nodes with <70% bootstrap support were collapsed. Branches are coloured according to clustering of the alphasatellites. Red: coconut foliar decay alphasatellites; orange: babuvirus alphasatellites (incl. CFDA3 and CFDA6); brown, ochre, green: alphasatellites associated with begomoviruses and those isolated from insects; olive, violet, blue: nanovirus-associated alphasatellites. Names are according to^25^ and GenBank accession numbers are indicated. (**b**) A pairwise DNA sequence comparison plot by SDT with % identity shown as a multicolour heat map^61^.

**Figure 3:**
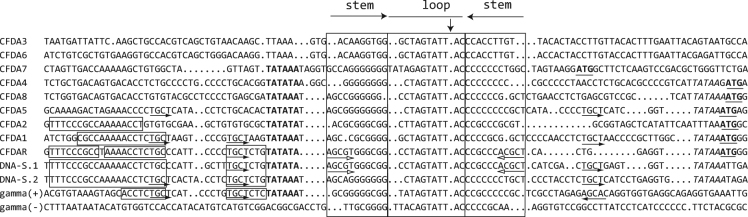
Comparison of replication origin sequences of 12 DNAs found associated with CFD disease. The origin sequences of components (indicated on the left) are aligned. Inverted repeat sequences (horizontal arrows) potentially forming a stem-loop (STL) are boxed. The vertical arrow indicates the position of potential cleavage by Rep protein. Conserved sequences shared by DNA-S.1, DNA-S.2 and some alphasatellites, are indicated by small boxes. The pentanucleotide AGCGT at the 5′ end of the stem-loop and its respective complement at the 3′ end shared by DNA-S.1 and CFDAR is indicated by an open-headed arrow, and the potential iteron sequence TGCT is indicated by an arrow. Potential TATA-box sequences at 5′ of the STL region are in bold; ATG start codons of *rep* genes, if within the borders of the alignments, are in bold and underlined; potential TATA-box sequences at the 3′ of the STL region are in italics. Gamma (+) and gamma (−) are two potential origin sequences that occur at different positions of the plus or the complementary strand of CFDV DNA-gamma.

All other 1.3 kb DNAs identified by cloning of RCA DNA products (Table S2) classify as alphasatellites (Fig. 2). One of them is, apart from two single nucleotide polymorphisms, identical with the molecule described previously^8^. Here we refer to it as coconut foliar decay alphasatellite 1 (CFDA1) since it was the first one discovered. All these alphasatellites as well as the Rep proteins encoded by them differ clearly from the master Rep-encoding DNAs (DNA-R) of the nanovirids (Fig. S1 and^12^). Although, based on the Rep protein comparisons, no obvious CFDV master Rep protein could be identified, we reasoned that, by analogy to the master Rep concept established for nanoviruses^23^, a master Rep-encoding DNA may have sequences in common with all other integral virus genome components, the replication initiation of which depends on the master Rep. We identified such common motifs shared by DNA-S.1, DNA-S.2 and one alphasatellite in the sequences flanking the inverted repeats that bracket the conserved nonanucleotide TAGTATTAC (Fig. 3). They are located 5′ of the inverted repeats and may contribute to origin recognition by a master Rep as shown for nanovirids^23,24^. We hence designated the alphasatellite that shares these common sequences with DNA-S.1 and -S.2 coconut foliar decay alphasatellite R (CFDAR), referring to the nanovirid DNA-R molecules^23^. In addition, only alphasatellite R and DNA-S.1 share the pentanucleotide AGCGT at the 5′end of the inverted repeat (5′ stem) and its respective complement at the 3′ end (Fig. 3). The other CFD-associated alphasatellites identified from CFD samples by direct cloning or by deep sequencing (Table 1) are numbered CFD alphasatellite 2 to 8 (CFDA2 – CFDA8).

Comparison of all CFD alphasatellites with selected babu-, nano-, and begomovirus-associated as well as whitefly- and dragonfly-associated alphasatellite species revealed that they form a clade distinct from other alphasatellites, except for CFDA3 and CFDA6, which group with the babuvirus alphasatellites (Fig. 2). Alphasatellite names are based on a recent taxonomy proposal^25^, and only representatives of the most distantly related alphasatellite species were compared.

To assess potential recombination among the CFD alphasatellites we used RDP4^26^ and detected one recombination event strongly supported by seven detection methods implemented in RDP4: the region from position 951 to 1099 in CFDA7 was identified as a possible recombinant with CFDA6. For details see Fig. S2.

**Figure 4:**
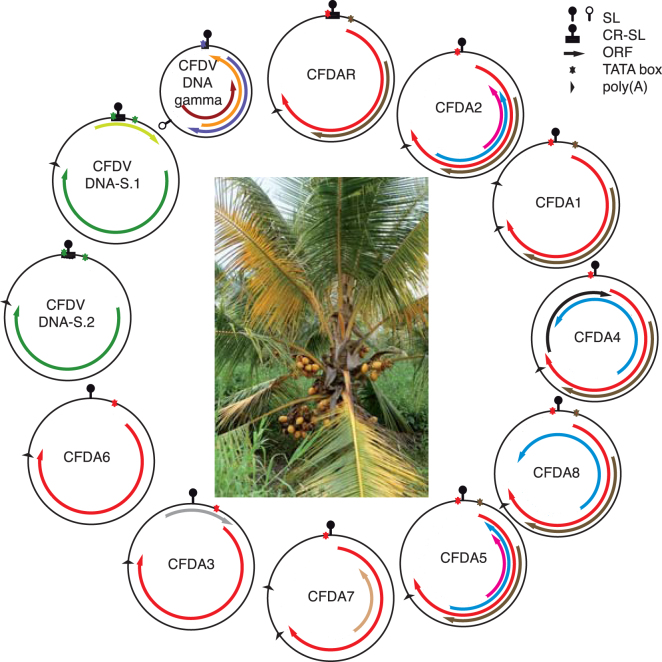
Symptoms of coconut foliar decay disease and genetic organisation of circular DNAs found associated with it. The identified DNAs are grouped according to their phylogenetic relationships. The presumed integral genome components DNA-S.1, DNA-gamma and alphasatellite R are shown next to each other. Inside each circle the name of the respective DNA is given. Shared potential open reading frames (ORFs) are indicated by the arrows of the same colours: red for the ORF encoding Rep (replication initiator protein); green for CP (capsid protein); brown for CFDAR_ORF2, CFDA1_ORF2, CFDA2_ORF2, CFDA4_ORF2, CFDA5_ORF2, CFDA8_ORF2; pink for CFDA2_ORF4 and CFDA5_ORF4, blue for CFDA2_ORF3, CFDA4_ORF4, CFDA5_ORF3 and CFDA8_ORF3. All other ORFs shown in different colours (orange, light-brown, grey, lime-green, purple, violet and black) are unique. SL – potential stem loop (inverted repeat sequences flanking the replication origin), symbolized by a knob; an empty knob represents the potential stem loop on the complementary (−) strand of CFDV DNA-gamma; CR-SL - common region around the stem loop region. Where TATA boxes could be associated with ORFs the colours of their indicative asterisks match those of the ORFs. In the centre, the CFD-affected MRD palm from which the 2015 sample originated is shown.

In addition to the CP-encoding DNAs S.1 and S.2 and the CFD alphasatellites, a different and smaller DNA was identified using rolling circle amplified DNA from CFDV virions (Table S2). The molecule of 641 nt has no similarity with any sequence currently in GenBank and bears ORFs in both orientations (Table 1 and Fig. 4). Curiously, this DNA has a second origin-like inverted repeat sequence at nts 396–423 flanking the nonanucleotide CAGTATTAC in (−) orientation (Fig. 3). There is no information about the polarity of the strand that is encapsidated or on the sequence that may act as replication origin. We named this molecule DNA-gamma. Apart from its size, DNA-gamma has no similarity with Old- and New World deltasatellites^27–29^. Figure 4 and Table 1 summarise common and distinctive features of DNAs derived from two CFDV samples of 1988/89.

Having found ten different circular DNAs of three types in virion-derived DNA of two samples from four different palms (for details see Table S1), we used specific primers to determine whether these DNAs could be also detected in samples prepared from ten additional symptomatic palms collected in 1988, 1989 and 2013 (Table 2). Only DNA-S.1, DNA-gamma and CFDAR were detected in each sample. Two combined samples of 1988 from three Vanuatu hybrid coconut palms contained all ten DNAs (Table 2).

**Table 2:** Identification of CFD-associated DNAs in different samples by PCR amplification.

Sample	1988–1989, palm ID/(sample No)^a^						2013^b^
DNA	cfd2 + cfd3 (3 + 6)	cfd4 + MRD25–21A (7 + 8)	MRD19 + MRD20 (9 + 32)	MRD37.14 (10 + 11)	MRD caged^c^(22 + 23)	TT17–7 + 12–86–14 + 12-86-15 (24 + 26)	one MRD palm, 11 leaves
CFDV DNA-S.1	+	+	+	+	+	+	+
CFDV DNA-S.2	−	−	+	−	−	+	−
CFDV DNA-gamma	+	+	+	+	+	+	+
CFDAR	+	+	+	+	+	+	+
CFDA1	+	−	−	−	−	+	+
CFDA2	−	−	+	+	−	+	−
CFDA3	+	−	+	−	−	+	−
CFDA4	+	−	+	−	−	+	+
CFDA5	+	−	+	−	−	+	−
CFDA6	+	−	+	−	−	+	−
CFDA7	−	−	+	−	−	+	−
CFDA8	+	−	−	−	−	+	−

^a^Two samples (indicated in  brackets) of virions prepared from one or several palms (indicated at the top; see Table S1) were pooled. DNA was subjected to RCA amplification followed by PCR using the specific primers indicated in Table S3.

^b^Total DNA of samples prepared from eleven individual symptomatic leaves of a single severely diseased palm were pooled and subjected to RCA amplification that was followed by PCR using specific primers.

^c^Caged plants experimentally infected by viruliferous *Myndus taffini*.

### Are there further CFD-associated DNAs sharing common sequences with DNA-S.1 and alphasatellite R?

Reasoning along the same lines that led to the discovery of the master Rep (M-Rep)-encoding DNAs of subterranean clover stunt virus (SCSV) and milk vetch dwarf virus (MDV)^23^ we compared CFDV DNA sequences flanking the inverted repeats that bracket the conserved nonanucleotide, the presumed replication origin, and uncovered stretches of sequence conservation (Fig. 3). We designed primers to amplify molecules with origin sequences common to DNA-S.1 and CFDAR to potentially identify CFDV DNAs other than DNA-S.1 and CFDAR (primers CFDV_STL1-dir and -rev; Table S3). PCR amplifications using DNA of samples CFD3 and CFD9 yielded 1.3 kb products that were cloned and sequenced. To avoid excessive redundant sequencing of the same DNAs, we screened the recombinant plasmids by PCR using CP ORF- and Rep ORF-specific primers (CFDV_S1-HindIII-dir and CFDAR-BamHI-dir, Table S3) in combination with the sequencing primers M13-dir and M13-rev. This way we analysed >100 recombinant plasmids. All 1.3 kb inserts represented either DNA-S.1 or CFDAR (data not shown). Similarly, we screened by PCR 60 additional recombinant plasmids containing 1.3 kb DNA, using primers CFDV_STL2-dir and -rev (Table S3), designed to potentially amplify CFDV DNAs with sequences common to the DNA-S.2 origin. No inserts other than DNA-S.2 were identified (data not shown).

Since targeting common origin sequences to identify a potential master Rep-encoding CFDV DNA failed, we searched for potential common amino acid motifs shared by the master Rep proteins and not by any alphasatellite Rep, a ‘master Rep signature’. Alignments of alphasatellite Rep sequences of CFDV, babu- nano- and begomoviruses in comparison to nanovirid M-Rep sequences indeed revealed several amino acid motifs characteristic of M-Rep proteins. We designed degenerate primers based on DNA corresponding to motifs EGP(W/F)E(F/Y)G and KNGI(I/V)QSGKY (Fig. S3, Table S3) and employed different combinations of these primers for PCR using DNA from samples CFD_1988/89 and CFD_2013. Total DNA prepared from banana bunchy top virus (BBTV)-infected banana leaves (BBTV-Hawaii_2013) and cloned faba bean necrotic stunt virus (FBNSV) DNA-R^30^ served as positive amplification controls. This way we obtained PCR products of the expected sizes from both BBTV and FBNSV substrates whereas no such PCR products were obtained from the CFDV samples (Fig. S4). Using DNA of the same CFDV samples and DNA-S.1-, DNA-gamma-, and CFDAR-specific primers we obtained PCR products of the respective expected sizes (Fig. S4).

Hence, attempts to find additional CFDV DNAs based on the master Rep concept^23^ did not uncover any.

### DNA replication assays in leaf discs

Replication initiation in *trans* of other genome components is the key feature to functionally distinguish nanovirid master Rep proteins from alphasatellite Rep proteins^23,24,31^. Although *Nicotiana benthamiana* is not an experimental host of nanoviruses, leaf discs derived therefrom have served for replication assays of nanovirus DNAs. We therefore tested whether any of the CFD alphasatellite-encoded Rep proteins were capable of initiating replication of their respective cognate DNAs or DNA-S.1, -S.2 or -gamma. We introduced redundant copies of these DNAs in the binary T-DNA vector pBin19 into agrobacteria and inoculated *N. benthamiana* leaf discs as described^31^. Whereas replication of FBNSV DNA-R was readily observed using that assay, we were not able to detect replicative forms of any of the CFDV DNAs (data not shown). Additional attempts to establish a CFDV DNA replication assay in leaf discs of *Hibiscus rosa-sinensis*, a relative of the potential alternative host *Hibiscus tiliaceus* of CFDV^7^, and hyacinth (*Hyacinthus orientalis*) as an example of a monocotyledonous plant, also failed.

### Do the CFD-associated DNAs identified here represent the CFDV genome?

In the absence of an infection assay for testing the cloned CFDV DNAs and being unable to prove replication of any CFD-associated DNAs in a leaf disc assay, we performed several deep sequencing experiments aimed at uncovering potentially missed CFDV DNAs. For that purpose RC-amplified DNA from twenty-three available virion samples (Table S1) was pooled to constitute sample CFD_88/89.

A total of 17,427,262 reads were obtained for the CFD_88/89 sample by Illumina HiSeq sequencing. From these, DNA-S.1, DNA-gamma, alphasatellite R, −2, and −4 could be *de novo* assembled. Moreover, one additional Rep-encoding DNA, CFDA5, was assembled *de novo*. DNA-S.2, CFDA1, CFDA3 and CFDA6 could only be assembled using sequences of the already available cloned CFDV DNAs as guides. No other circular DNAs sufficiently covered by highly abundant reads were detected. Therefore all reads were mapped to the sequences of the cloned molecules using Geneious. This way, 13,218,631 reads (about 76%) could be mapped to the previously identified sequences (Table 3). Average coverage ranged from a depth of 1,355,773 × for the very abundant DNA-gamma (54% of all reads) to a depth of 3,361 × for CFDA4 (0.25% of reads). DNA S.1 and CFDAR matches were equally abundant (~6.1% of reads). Reads not matching CFDV sequences were reassembled and checked for the presence of known virus sequences by BLAST: no matches of significant abundance with any ssDNA- or other virus-like sequences were found. These results led us to conclude that we had identified all CFD-associated sequences of significant abundance in the 1988/1989 samples.

**Table 3:** Summary of deep sequencing data from three CFD samples.

Sample	CFD_88/89^a^ (17,427,262 total reads)			CFD_2013^a^ (23,513,930 total reads)			CFD_2015^b^ (9,736,564 total reads)		
DNA^c^	Reads	% of reads	Cov. Mean	Reads	% of reads	Cov. Mean	Reads	% of reads	Cov. Mean
CFDV DNA-S.1	1,080,158	6.19	80,671	208,259	0.88	15,535	113	0.001	15
CFDV DNA-S.2	74,215	0.43	5,608	(212)^d^	0.01	8	228	0.002	32
CFDV DNA-gamma	9,413,561	54.01	1,355,773	3,921,211	16.67	569,975	88	0.001	20
CFDAR	1,060,676	6.07	84,048	214,390	0.91	16,342	90	0.001	12
CFDA1	56,466	0.32	4,232	9,811,837	41.72	741,366	0	0	0
CFDA2	623,396	3.58	47,905	(342,452)	1.46	32,470	14	<0.001	2
CFDA3	179,488	1.02	14,836	0	0	0	0	0	0
CFDA4	44,202	0.25	3,361	85,965	0.36	6,482	0	0	0
CFDA5	50,694	0.29	3,789	(11,327)	0.05	458	0	0	0
CFDA6	354,325	2.03	27,703	0	0	0	0	0	0
CFDA7	256,500	1.47	19,862	0	0	0	0	0	0
CFDA8	24,950	0.14	1,876	(3,618)	0.02	139	317	0.003	42
total	13,218,631	75.85		14,599,273	62.08		850	0.008	

Geneious mapping of total reads against all 12 identified CFD associated DNAs, performed with the settings allowing 10% mismatch per read. Cov. Mean – average coverage.

^a^DNA deep sequencing;

^b^RNA deep sequencing;

^c^GenBank accession numbers of DNAs used for mapping are the following:

CFDV DNA-S.1 - MF926436, CFDV DNA-S.2 - MF926439, CFDV DNA-gamma - MF926441, CFDAR - MF926434, CFDA1 - MF926424, CFDA2 - MF926426, CFDA3 - MF926427, CFDA4 - MF926429, CFDA5 - MF926430, CFDA6 - MF926431, CFDA7 - MF926432, CFDA8 - MF926433.

^d^Brackets indicate ambiguous mapping due to sequence similarity with other DNAs; only a small part of the component was covered.

In the same way a total of 23,513,930 reads was obtained (Illumina HiSeq) using RC-amplified templates from total DNA (non virion-encapsidated) prepared from symptomatic leaves of 2013. About 62% of the reads (14,599,273) could be mapped to the available CFDV sequences, with DNA-S.1, CFDAR, DNA-gamma, CFDA1 and CFDA4 being completely covered. DNA-S.2, CFDA2, CFDA5 and CFDA8 had incomplete coverage and CFDA3, CFDA6 and CFDA7 had no matches (Table 3). Only the five DNAs that were completely covered by mapping, DNA-S.1, CFDAR, DNA-gamma, CFDA1 and CFDA4, could also be identified by PCR using component-specific primers on RC-amplified DNA from the 2013 sample pool.

To show that all DNAs of a multicomponent ssDNA virus could be detected in the same way that we used for analysis of the CFD-associated DNAs, we amplified and sequenced DNA from BBTV-infected leaves from Hawaii, Nigeria and Vietnam. All BBTV genome components could be *de novo* assembled, representing ~92% of ~26.8 million reads for the sample from Hawaii, ~52% of ~25.86 million reads for the sample from Nigeria and 52% of 34.5 million reads for the sample from Vietnam (Table S4). In addition, a variant of the proposed alphasatellite species banana bunchy top alphasatellite 2^25^ was identified in the last sample.

Furthermore, we were able to *de novo* assemble CFDV sequences using Illumina RNA-Seq technology^32^ and total RNA from a diseased MRD coconut palm leaf (Fig. 4) sampled in 2015 on Espiritu Santo, Vanuatu. From 9,736,564 merged paired end reads, DNA-S.2 could be *de novo* assembled (differing in 3 single nucleotides from the sequence of the cloned DNA-S.2 from the CFD_88/89 sample). Also DNA-S.1, DNA-gamma and CFDAR could be assembled using the already available CFDV sequences as references (Table 3, CFD_2015 sample). A new alphasatellite, CFDA8, was identified in this sample. Interestingly, CFDA8 (differing by 11% from the CFDA4 sequence) could also be identified in the DNA reads of the CFD_88/89 sample pool by guided assembly, yet with lowest average coverage and read count (Table 3). We then verified the presence of CFDA8 in the 1988/89 and 2013 samples by PCR amplification using component-specific primers (Table 2). Considering the sequence variation of the CFD associated DNAs between 1988/89 and 2013 or 2015 we determined average nucleotide substitution rates of 6.3 × 10^−4^/site/year for CFDA1 and of 9.4 × 10^−5^/site/year for CFDAR and CFDV DNA-S1/-S2. Both are within the range of those determined for other ssDNA viruses^33,34^ but clearly lower than that of nanoviruses^35^ (for number and types of nucleotide changes see Table S5). No sequence variation over a time span of 26 years was observed for CFDV DNA-gamma.

Applying high throughput DNA and RNA sequencing to search for CFD-associated DNAs, only the three types of molecules identified earlier were uncovered: alphasatellites, DNA-S and DNA-gamma. Two new and distinct alphasatellites (CFDA5 and CFDA8) were first found by deep sequencing. Subsequently their physical presence in several samples of 1988/89 was proven by PCR and cloning. Hence, deep sequencing and amplification using component-specific primers yielded consistent data about the presence or absence of a given CFD-associated DNA in the samples analysed (Table 2). No sequences of other ssDNA- or RNA viruses and viroids were identified in DNA and RNA from CFD-affected palms.

## Discussion

CFD, first described around 1964^2,36^, was further characterized during the following decades^1,7,37^ and a virus (CFDV) with isometric particles and one ssDNA molecule of 1291 nt was uncovered^3,8,38^. The CFD pathosystem still lacks essential information about the virus, plant hosts of vector and virus, and the environment^39^. Therefore, our objective was to better characterise the DNAs associated with CFD.

Applying RCA to DNA of CFDV particle samples archived in 1988/89 we cloned and identified nine different circular DNAs of 1252 to 1291 and one of 641 nucleotides, respectively (Table S2). Whereas the 1988/89 samples used for cloning and deep sequencing originated from a total of ten different palms, the 2013 samples were from a single palm only, which may explain the lower diversity of CFD-associated DNAs therein. Two of these (DNA-S.1 and DNA-S.2) encode the capsid protein, suggested by its deduced size of 24 kDa, which fits well with that observed in virion preparations by SDS-PAGE, and  was confirmed for CP1 by mass spectrometry (Fig. 1). The fact that DNA-S.2 was not detected in all samples indicates that it may represent a variant CP-encoding DNA rather than the genome component of a second CP. Remarkably, DNA-S.2, the gene of a variant virion, was maintained in the virus population from 1989 through 2015. CFDV CP has about equal sequence similarity (19–24%) to that of DesMoV, an Old World begomovirus infecting legumes^22^, and that of the grapevine-infecting grabloviruses^20,21^, which characterizes it as a sort of intermediate type of CP with elements of aleyrod- and cicadellid-transmitted viruses. It is interesting to note that CFDV is transmitted by the planthopper *M. taffini*^1,37^ and grabloviruses by treehoppers (e.g. *Spissistilus festinus* Say)^40^. Their capsid protein similarity may reflect the relationship between the vector insect families. All these findings suggest a taxonomic position of CFDV outside the family *Nanoviridae*. Despite its capsid protein sequence similarity with certain geminiviruses, the available genome sequence information, the particle morphology and the capsid protein size suggest that CFDV is not a member of the *Geminiviridae*.

All ssDNA viruses require a replication initiator protein, Rep, encoded either by the cognate DNA that also encodes the capsid protein or by a separate DNA. The latter is the case for nanovirids where a master Rep protein serves that function. In addition to a virus-specific DNA-R molecule, varying numbers of Rep-encoding alphasatellites associate with babu-, nano- and geminiviruses^25^. Alphasatellite Rep proteins are distinct from nanovirus master Rep proteins (Fig. S1 and^12^). The fact that we found only alphasatellite-like DNAs associated with CFD, raises the following questions: (i) is there a master Rep-encoding DNA that has not been identified yet, or (ii) may one (or several) of the CFD alphasatellite-encoded Rep proteins act as master Rep for the other CFDV DNAs?

Unlike nanovirid-associated alphasatellites a DNA-R shares sequences near the replication origin with all integral nanovirid DNAs the replication of which depends on M-Rep action^23^. Only a subset of CFD alphasatellites shares such sequences with DNA-S.1 and DNA-S.2, i.e. CFDAR, and partly CFDA1 and CFDA2 (Fig. 3). Also the small CFDV DNA-gamma has two short iteron-like sequences 5′ of the origin inverted repeat in common with CFDAR and DNA-S.1 and -S.2 (Fig. 3). The fact that only CFDAR and DNA-S.1 share the pentanucleotide ACGCT at the 5′end of the inverted repeat (5′ stem) or its respective complement at the 3′ end adds another element in common between these two DNAs. Among the few physical binding studies of ssDNA virus Rep proteins with sequences of the origin region this part of the potential stem-loop sequence has been shown to interact *in vitro* with the purified porcine circovirus Rep endonuclease domain^41^. Also the fact that CFDAR was the only alphasatellite identified in all CFD samples favours our speculation that it encodes a Rep protein required for replication initiation of DNA-S.1, -S.2 and DNA-gamma and may act as master Rep for CFDV.

For the CFDA1 Rep protein expressed in *E. coli* or yeast, biochemical data have shown that its DNA-binding properties, oligomerization and ATPase activities are comparable to those of geminivirus or nanovirid Rep proteins^42^. However, CFDA1 replication assays in a variety of non-host cell protoplasts or bombarded cells failed^42^. We also failed to establish a replication assay for CFD-associated DNAs in leaf-disc tissue of three different plant species. The reason for this replication failure in non-host cells of CFDA1 and other CFDA molecules tested by us remains unknown. The CFDA1 Rep promoter is phloem specific as has been shown in tobacco protoplasts and transgenic plants^43,44^. The phloem-specificity is in agreement with the phloem limitation of the virus in coconut palms^6^. Strict phloem-specificity of CFDV promoters could be one reason for the unsuccessful attempts to establish replication in non-host and/or non-phloem cells. Without a replication assay for CFD-associated DNAs the question of whether there is a single M-Rep or several Rep proteins that initiate replication of the CP-encoding DNAs and the 641 nt DNA-gamma remains unanswered.

In addition to the two types of ~1.3 kb CFD-associated DNAs, the CP-encoding DNA-S.1 or -S.2 and several Rep-encoding alphasatellites, we uncovered the abundant 641 nt DNA, DNA-gamma. Molecules similar in size compared to DNA-gamma were found associated with geminiviruses, such as tomato leaf curl virus (ToLCV)^45^ and sweet potato leaf curl viruses^27–29^. These deltasatellites contain an A-rich region and a satellite conserved region or traces thereof^46^. CFDV DNA-gamma has no similarity with these deltasatellites and lacks an A-rich or satellite conserved region but, like the tomato leaf curl deltasatellite (ToLCD), it has a second potential stem-loop sequence^45^. It bears small ORFs on each strand with respective coding capacities for small proteins of 9.5 kDa (plus polarity) and 11.5 or 11.1 kDa (minus polarity), respectively. There is no significant similarity with sequences in GenBank. Given the apparent very high abundance of DNA-gamma in CFD-affected palms and its presence in all samples tested we consider DNA-gamma to be an integral component of the CFDV genome. The fact that DNA-gamma is almost precisely half the size of the other CFD-associated DNAs probably reflects the packaging constraints of the CFDV capsid: a DNA-gamma dimer would easily become encapsidated. Dimeric ssDNA forms are frequent in geminiviruses^47^ and probably in other circular ssDNA viruses, too. Also for BBTV smaller than canonical genome components have been described, but these were clearly defective molecules, mostly of DNA-R and DNA-R recombinants with other genomic DNAs; only a few of them were half the canonical size of about 1.1 kb^48^.

Plant viruses encode movement proteins to mediate their movement across plasmodesmata, for a review see^49^. ssDNA plant viruses also encode movement proteins, yet there are recent examples of grass-associated ssDNA viruses devoid of distinct movement proteins^50^. However it is not clear whether the members of the *Genomoviridae* are genuine plant-infecting viruses or rather have grass-associated fungi as hosts as for instance Sclerotinia sclerotiorum hypovirulence-associated DNA virus 1^11^. The genome organization of genomoviruses (monopartite, Rep- and capsid proteins encoded on opposite strands, divergent transcription) clearly differs from the CFD-associated DNAs.

**Figure 5:**
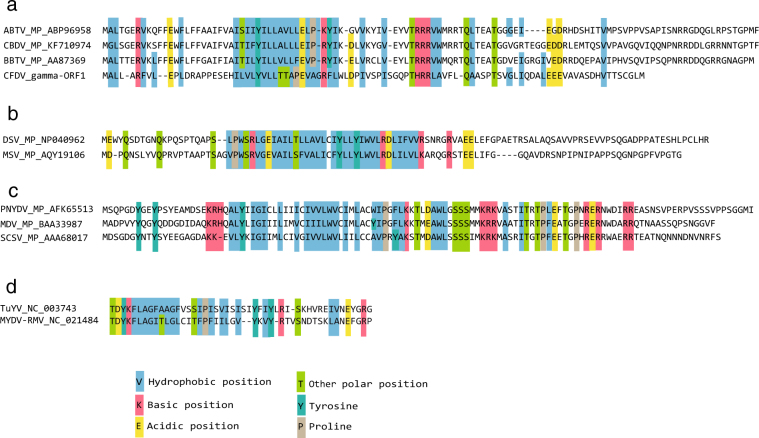
Alignment of amino acid sequences of movement proteins of several ssDNA viruses and luteoviruses. Alignments were done using ClustalW in MegAlign of DNASTAR. Names and accession numbers of the movement proteins of selected babuviruses (**a**), mastreviruses (**b**), nanoviruses (**c**) and luteoviruses (**d**) are indicated on the left. Potential movement protein ‘signature’ – stretches of hydrophobic amino acids as well as of basic (R, K) and acidic (D, E) side chains are boxed. The similarity of amino acid stretches of the potential ORF 1 protein of CFDV DNA-gamma is highest with the movement proteins of babuviruses.

To the best of our knowledge, no generally conserved sequence motifs were described for plant virus movement proteins, but one may notice some limited amino acid similarity: stretches of hydrophobic amino acids surrounded by basic (R, K) and acidic (E, D) amino acids (Fig. 5). We observed these sequence signatures in movement proteins of nanovirids and geminiviruses, as well as in a recently described small movement protein (P3a) of luteo- and poleroviruses^51^. There is a curious similarity of the above mentioned amino acids in the deduced ORF1 protein of DNA-gamma at a position corresponding to similar amino acids in the movement proteins of babuviruses (Fig. 5a). We speculate that DNA-gamma may encode the CFDV movement protein.

Are these three types of DNAs the CFDV genome? Only infection assays fulfilling Koch’s postulates will provide final proof. In the absence of such, how confident can we be to have identified all CFDV genome components? Three deep sequencing experiments employing encapsidated DNA from virions, total DNA and total RNA from diseased palms confirmed what we had found earlier by RCA and cloning. Only two additional alphasatellites were uncovered this way and subsequently confirmed by PCR and cloning. The fact that for virion-derived DNA 75% of about 17.4 million deep sequencing reads represented CFDV sequences and 62% of the total DNA-derived 23.5 million reads represented CFDV sequences (Table 3) shows that enrichment of CFDV sequences was efficient. Furthermore, having been able to *de novo* assemble a CFDV DNA-S.2 molecule from 0.002% total RNA reads (219 of about 9.7 million reads; Table 3) illustrates the sensitivity of our searches. Moreover, the deep sequencing of RNA, which allows detection of RNA and DNA viruses^52^, did not uncover any RNA virus associated with CFD.

Among the multitude of circular replication-associated protein encoding single-stranded (CRESS) DNA viruses there is an example of a somewhat similar case to CFDV, the Pacific flying fox faeces associated multicomponent virus-1 (PfffaMCV-1)^53^. The virus has a Rep-encoding DNA, a CP-encoding DNA, and a DNA encoding a potential protein with no similarity to any protein in the database. Like CFDV DNA-S.1, CFDV DNA-gamma and CFDAR these three PfffaMCV-1 DNA types share only common sequences flanking the inverted repeat and the nonanucleotide at the replication origin. Hosts of this virus are not known.

Recently, a complex of a nanovirus and fourteen alphasatellites was described from *Sophora alopecuroides*^14^. In the case of sophora yellow stunt associated virus (SYSaV), five distinct M-Rep-encoding DNAs and two Clink-encoding DNAs were found along with the respective other genome components. No data on the role of the five M-Rep-encoding DNAs in this nanovirus alphasatellite complex were reported. Despite the fact that alphasatellites are found associated with an increasing number of geminiviruses and nanovirids^14,15^, only few data are available about their potential role for the biology of the viruses they associate with. There is a report about disease symptom attenuation by an alphasatellite in combination with tomato yellow leaf curl virus from Oman, a monopartite Old World begomovirus^54^, possibly by interfering with the accumulation of the tomato leaf curl betasatellite that enhances the pathogenicity of the virus. An opposite effect, aggravation of disease symptoms was recently reported for an alphasatellite associated with euphorbia yellow mosaic virus from Brazil, a bipartite New World begomovirus^55^. By contrast, virus transmission by the whitefly vector *Bemisia tabaci* was negatively affected. Given the number of different alphasatellites associated with CFD, any effect on the disease caused by the differential presence or absence of alphasatellites in individual trees is easily conceivable. Such a differential presence of alphasatellites might be the origin of a peculiar phenomenon of disease remission reported for differently inoculated palm trees^56^.

Given the three types of circular ssDNAs associated with coconut foliar decay, the capsid protein encoding DNA-S.1 and -S.2, DNA-gamma, half the size of DNA-S, and the multitude of alphasatellites including one that may substitute for a canonical nanovirid DNA-R, it appears that CFDV may represent an ancient lineage of ssDNA viruses or a re-assorted virus with features of nanovirids (virion morphology, segmented genome) and geminiviruses (vectored by a planthopper, capsid protein relationship). The fact that CFD has so far only been found in Vanuatu, a rather isolated archipelago, may have contributed to the evolution and conservation of such a unique combination of ssDNAs yielding CFDV, a truly ‘in-between’ virus.

## Methods

### Virus isolates and virion preparations

Three different sources of CFD-associated virus or nucleic acid were used in this study: (i) purified virions, (ii) total DNA extracts and (iii) total RNA extracts prepared from diseased coconut palms. Leaves were harvested from naturally infected symptomatic coconut palms at the Vanuatu Agricultural Research and Training Centre (VARTC), Espiritu Santo, Vanuatu. Virus was partially purified from leaves collected in 1988 and 1989, air-freighted at ambient temperature to Adelaide and stored at −20 °C. Aliquots of virion preparations^38^ were stored in 50% glycerol at −80 °C. Ten μl samples buffered in 20 mM Tris, 2 mM EDTA, pH 8, were shipped at ambient temperature to France in 2012. Virion preparation CFD3 (sampled in November 1988) was from pooled leaves of two symptomatic hybrid coconut palms and preparation CFD9 was from two palms of the highly susceptible variety ‘Malayan Red Dwarf’ (MRD), sampled in September 1989. Details of the collection dates and the origin of different virion samples prepared in 1988 and 1989 are described in Table S1. In addition, total DNA extracts were prepared from eleven leaves harvested from one MRD palm in 2013, and total RNA extracts were from leaves harvested from a single MRD palm diseased in 2015.

Leaves from banana plants (*Musa* spp.) infected with BBTV were harvested in Hawaii, Nigeria and Vietnam in 2013 and total DNA was extracted to produce the BBTV-Hawaii_2013, BBTV-Nigeria_2013 and BBTV-Vietnam_2013 samples.

### Capsid protein identification

Virions prepared from the 1.18–1.23 g/ml density zone of an isopycnic Nycodenz gradient^38^ were subjected to a second isopycnic density gradient centrifugation in caesium sulphate. Fractions containing the viral DNA (1.29–1.32 g/ml) were pooled and denatured prior to analysis by 3–13% discontinuous SDS-polyacrylamide gel electrophoresis (SDS-PAGE)^57^. Proteins were detected by silver staining, and the M_r_ of the capsid protein determined by comparison with co-electrophoresed marker proteins.

For identification by LC-MS/MS virions of ten samples collected in 1988 and 1989 (samples 2, 4, 5, 6, 7, 8, 10, 22, 23 and 29, Table S1) in which no DNA-S.2 was detected, were pooled, denatured and proteins separated by 15% SDS-PAGE. After staining by Coomassie brilliant blue the major protein band of ~24 kDa was excised, digested by trypsin and analysed by nanoLC-MS/MS as described^58^. Protein identification was performed using the Mascot database search engine (Matrix Science, London, UK) against the CFDV-CP1 sequence with trypsin specificity and two missed cleavages. Fixed and variable modifications included carbamidomethylation of cysteine and oxidation of methionine, respectively. Peptide and fragment tolerance were respectively set at 15 ppm and 0.05 Da. Only peptides with Mascot ion scores above identity threshold (20) at less than 1% FDR (false discovery rate) were considered.

### Nucleic acid extracts

Total DNA from plant tissue was extracted according to a modified Edwards protocol as described previously^30^. Total RNA was prepared from plant tissue by GenCatch Plant RNA Purification Kit (Epoch Life Science).

### Rolling circle amplification (RCA), cloning and sequencing

RCA was done on total DNA extracted from plant tissue or directly on virion samples with the Illustra TempliPhi Amplification Kit (GE Healthcare). Individual virion samples or samples of total DNA extracts from diseased coconut palm leaves and banana leaves were diluted 10-fold in 5 mM Tris-HCl, pH 7.6, one-μl aliquots were mixed with 5 μl of sample buffer, denatured at 95 °C for 3 min, then chilled on ice before adding 5 μl of reaction buffer and 0.2 μl of the Phi29 DNA polymerase. Incubation was for 20 h at 30 °C followed by 10 min at 65 °C. For CFDV samples, RCA products were digested with various restriction enzymes in appropriate buffers, and the fragments generated by *Aat*II, *Eco*RI, *Bam*HI, *Kpn*I, *Age*I and *Sal*I were resolved in 1% agarose gels, extracted and inserted either into plasmid Litmus28 (New England Biolabs) or pBluescript KSII (+) (pBKSII) (Stratagene). Candidate bacteria harbouring recombinant plasmids were analysed for the presence of the insert by colony-polymerase chain reactions (PCR) using M13- direct and M13-reverse primers, as described previously^30^. To avoid repetitive redundant sequencing of CFDV DNAs, the inserts were amplified by PCR using M13 direct and reverse primers, and the PCR products were subjected to restriction fragment length polymorphism (RFLP) analysis: 10 µl of colony-PCR products were digested with *Hae*III or *Sau*3A restriction endonucleases in 15 µl of the appropriate buffer and resolved in 1.5% agarose gels. Recombinant plasmids were grouped according to their digestion patterns. Insert DNAs of at least three plasmids, each representing a distinct RFLP pattern, were sequenced. The inserts in the recombinant plasmids were Sanger sequenced at GATC Biotech (Konstanz, Germany). To confirm that the sequences obtained from the cloned restriction enzyme-generated DNAs represented a complete circular component, specific respective back-to-back primers were used in PCR on RCA DNA of CFD samples. The amplified DNAs were inserted into *Hinc*II- or *Eco*RV-linearized plasmids and sequenced.

### PCR amplification of CFDV DNAs

RCA products were diluted ten-fold and one µl used to amplify CFDV DNAs by PCR, employing either Taq II DNA polymerase (Eurobio) or high fidelity Phusion DNA polymerase (Finnzymes), following the manufacturer’s instructions. PCR primers are listed in Table S3; annealing temperatures were varied according to the primer sequence.

### Deep sequencing

Two CFD associated DNA samples were prepared for deep sequencing. Individual RCA products obtained from virion preparations from samples 3, 7, 9, 11, 22 and 26 collected in 1988 and 1989 (Table S1) were pooled and designated sample pool CFD_1988/89. Individual RCA products obtained from total DNA extractions of 2013 from leaves 3, 5, 7, 8 and 10 (all leaves from one symptomatic palm) were pooled to yield sample pool CFD_2013. Individual RCA products obtained from total DNA extractions of three BBTV samples, BBTV-Hawaii_2013, BBTV-Nigeria_2013 and BBTV-Vietnam_2013, were also subjected to deep sequencing.

Barcoded sequencing libraries were prepared from 1 ng of RCA-enriched DNA for each sample using the Nextera XT DNA Library Prep kit (Illumina, San Diego, CA, USA), following the manufacturer's instructions. Libraries were paired-end sequenced (2 × 101 bp) on an Illumina HiSeq. 2000 DNA sequencer (Illumina, San Diego, CA, USA) at ANU, Canberra.

Total RNA prepared from leaves of a symptomatic coconut palm harvested in 2015, designated as CFD_2015 sample, was used for RNA deep sequencing. RNA sequencing was performed using Illumina RNA-Seq technology as described previously^32^. Briefly, from total RNA (see nucleic acid extracts) the ribosomal RNA was physically subtracted using a RiboMinus Plant Kit according to the manufacturer’s protocol (Life Technologies), and the resulting RNA served as the template for cDNA synthesis with random octamer primer using Revert Aid H Minus Reverse Transcriptase (Thermo Fisher Scientific). After a clean-up step second strand synthesis was done using the NEBNext mRNA Module (New England BioLabs Inc.). The sequencing library was prepared with the Nextera XT Library Kit (Illumina) and run after quality check on Illumina MiSeq as paired end reads (2 × 301 bp) (DSMZ, Germany).

### Sequence analysis

Sequences of cloned CFD DNAs were analysed with DNASTAR Lasergene, (v 12.1) (DNASTAR, Inc., Madison, WI). Consensus sequences of a given CFD DNA component were derived from at least three sequences of cloned DNA. Primary contigs (nodes) assembled by SPAdes^59^ from the Illumina reads were further assembled using SeqMan Pro of DNASTAR or Geneious (v 8). Guided assemblies were done with SeqMan Pro, Geneious and CLC Genomics Workbench software (v 8.0). Since SPAdes had produced too many obviously misassembled contigs (only DNA-gamma and DNA-S.1 could be *de-novo* assembled from the SPAdes produced contigs), the single reads (17,427,262 from the CFD_1988/89 sample and 23,513,930 from the CFD_2013 sample) were directly assembled into contigs using Geneious v10.2.3. In the same way all single reads were mapped against the sequences of the cloned RCA products using Geneious v10.2.3.

Unmapped reads (Geneious or CLC) were reassembled, and the resulting contigs were checked by BLAST for known virus sequences and obvious contamination by plant, microbe or human sequences.

Phylogenetic analyses were conducted in MEGA7^60^ and SDT v. 1.2^61^. Multialignments were done using Muscle^62^ as implemented in Geneious or MEGA7. A maximum likelihood tree (100 bootstrap repetitions) was constructed by PhyML^63^ with the GTR + G + I substitution model, chosen after model test in Mega7. BBTV DNA-R (GenBank acc. no. S56276) served to root the tree, and nodes with less than 70% bootstrap support were collapsed using Dendroscope, v3.5.8^64^. The final graphics were done in FigTree v1.4.3. (http://tree.bio.ed.ac.uk/software/figtree/). A recombination analysis was carried out using the recombination detection package RDP4^26^.

### CFDV replication assays in plant leaf discs

Viral DNA replication was assayed in leaf discs of *Nicotiana benthamiana, Hibiscus rosa-sinensis* (var. Valencia) and *Hyacinthus orientalis* essentially as described previously^31^. For replication assays, redundant copies (direct repeats) of CFDV components were constructed in the binary vector pBin19 (see Table S6 for construction details) and transferred into *Rhizobium radiobacter*, formerly *Agrobacterium tumefaciens*, strain LBA 4404 by electroporation. Six days post inoculation total DNA was isolated from the leaf-disc tissue, fractionated on 1% agarose gels and transferred onto Hybond-N membrane (Amersham). Viral DNA replicative forms were identified by Southern hybridization. Cloned CFDV DNAs served as templates for radioactive probes. Random-primed probe labelling with [α-^32^P]-dCTP (Perkin Elmer) was carried out with a DNA labelling kit (ICN Pharmaceuticals). Hybridization was conducted essentially as described previously^31^.

### Data availability

The sequences of all CFD-associated DNAs determined here are deposited in GenBank under the accession numbers MF926423 to MF926444.

## Electronic supplementary material

Supplementary Information
