# Supplementary material for: Analysis of DNAs associated with coconut foliar decay disease implicates a unique single-stranded DNA virus representing a new taxon
Source: Sci Rep. 2018 Apr 9;8:5698. doi: 10.1038/s41598-018-23739-y (PMC5890292; doi:10.1038/s41598-018-23739-y)
Supplement: Supplementary file 1 — Supplementary Information [file 41598_2018_23739_MOESM1_ESM.pdf]

**Supplementary Information**

# Analysis of DNAs associated with coconut foliar decay disease implicates a unique single-stranded DNA virus representing a new taxon

Bruno Gronenborn[1], John W. Randles[2], Dennis Knierim[3], Quentin Barrière[1], H. Josef Vetten[4], Norman Warthmann[5], David Cornu[1], Tiata Sileye[6], Stephan Winter[3], Tatiana Timchenko[1,*]

[1]Institute for Integrative Biology of the Cell, UMR9198, CNRS, Université Paris-Sud, CEA, Avenue de la Terrasse, 91198 Gif sur Yvette, France.
Phone: (+33) 16982 3468
Email: tania.timtchenko@i2bc.paris-saclay.fr
Email: bgronenborn@gmail.com
Email: quentin.barriere@i2bc.paris-saclay.fr
Email: David.CORNU@i2bc.paris-saclay.fr

[2]School of Agriculture Food and Wine, University of Adelaide, Glen Osmond, South Australia 5064.
Email: john.randles@adelaide.edu.au

[3]Deutsche Sammlung von Mikroorganismen und Zellkulturen GmbH (DSMZ) Messeweg 11/12,
38104 Braunschweig
Germany
Email: dek13@dsmz.de
stephan.winter@dsmz.de

[4]Im Spargelfeld 1
38162 Cremlingen
Germany
Email: rvetten@gmx.de

[5]Norman Warthmann
Research School of Biology
The Australian National University
Linnaeus Way, Canberra, ACT 2601, Australia
Email: norman.warthmann@anu.edu.au
norman@warthmann.com

[6]Vanuatu Agricultural Research and Technical Centre, Santo, Vanuatu
Email: tiatas@vanuatu.com.vu
sileye.tiata@gmail.com

[*]***Corresponding author***.

**Figures S1, S2, S3, S4; Tables S1, S2, S3, S4, S5, S6, S7**

## Figure S1

```
                                   ---MAS--SRRWCFTLNFS-AXEP---LSPLSENE-VSYAVWQDEXAPTTGQRHLQGYLQLK-KRT-LGGXKKLFGG--AHLEKARGTD-EEARDYCMKEETRVSGPWEFGEPCPSGSHKR----KLMERVIRSPEEMXXEDPSTYRRCKAKL--AEEEFXK---EEIWPXXLKPWQLEL
                                            10        20        30        40        50        60        70        80        90       100       110       120       130       140       150       160       170       180
CFDV-[VU;89]_alphasat_R_Rep        ---MGGR-ARRWCFTLNYSAEEEAASLVRRIESLN-TVYAIVGDEVAPTTGQRHLQGFLHLKGAGRTLQGLKTFLQNDKIHLERAKGSD-EQNRGYCSKE-QVRF---EHGVATRPGVRRG-----VRQRYVEDPDELRLEDPGGYRRCVASG--SSLEWARWALEHPFPHAYHDWQREL 163
CFDV-[VU;89]_alphasat_1_Rep        ---MGSS-IRRWCFTLNYETEEEAANVVRRIESLN-LVYAIVGDEVAPSTGQRHLQGFIHLK-TGRRLQGLKTVLGNDRIHLEPTRGSD-EQNRDYCSKE-RVLL---EHGVPTRPGVKRP----RLAQRFAEEPDELRLEDPGGYRRCVVHG--ASVEWTRWAAENPFPFPYHNWQLEV 163
CFDV-[VU;89]_alphasat_2_Rep        ---MAAR-VRRWCLTLNYSTEEEAARFVERVKSLT-TTYCIVGDEVAPTTGQHHLQGFIHLK-SARCLQGLKTFLQNDKVHLEAAKGSD-EQNRVYCSKE-QIRY---EHGVATRPGSKRR-----LEQRFDEDPADLRLEEPGGYRRVVAHR--ASVEWYGWALEHPFPHPYHDWQLEL 162
CFDV-[VU;89]_alphasat_4_Rep        ---MTQR-CRRWCFTLNYDSDEEAAALVRRVQSLS-LTYAIVGKETAPTTGQKHLQGFLHLKGGGRVLQGLKTVLQNDRIHLEQAHGSD-EQNRVYCSKE-ETIF---EHGIPTRPGVKRR-----LVDRFDDEPDELRLEDPGGYRRCVALR--AAVEWAGWASENPFPFPYHHWQLEL 163
CFDV-[VU;15]_alphasat_8_Rep        ---MAQR-XRRWCFTLNFNSEEEAAAVVRRLQSLS-LTYCIVGKETAPTTGQQHLQGFLHLKSGGRTVQGLKTVLQNDRIHLEQAHGSD-EQNKIYCSKD-EVIF---EHGIPTRPGVKRR-----LADRFDDDPDEVRLEDPGGYRRCVALR--SSVEWAGWAAENPFPFPYHHWQLEL 163
CFDV-[VU;89]_alphasat_5_Rep        ---MSSR-ARRWCFTLNYRSEEEAAQLVQRIESLD-PTYAIVGDETAPTTGQRHLQGFIHLKGAGKSLQALKTALRNDTVHLERAQGSD-LQNRDYCSKE-QVRF---EHGIATRAGCKRR-----LLERFAEDPDDLLLEDPGGYRRCVAHR--HAVEWREWATARDFPYPLHDWQHEV 163
CFDV-[VU;89]_alphasat_3_Rep        -MAMVS---RRWCFTLNYSEEDERTRLLSLFSEEE-LHYAIVGDEVAPSTGQKHLQGYLNFR-KVLRLGALKKKY-SDKAHWEIAKGTD-EENRVYCSKE-HKVF---ELGSPVVVGSNKR----KLAEAIERSPERMRLEQPEIFHRYASAK--KMIQFK---EQYDHPVFDRTWQIKL 159
CFDV-[VU;89]_alphasat_6_Rep        -MALQS---RRWCFTLNYSEEEERTRLLSLFSEEE-FHYAIVGDEVAPSTGQKHLQGYLNFR-KVLRLGALKKKY-SDKAHWEIAKGTD-EENRVYCSKE-HKLL---ELGSPVLVGSNKR----KLAEAIERSPERMRLEQPEIFHRYASAK--KMIQFK---EQYDHPVFDRTWQIKL 159
CFDV-[VU;89]_alphasat_7_Rep        ---MASQ-VRRWVLTFNYSDESAASDLVRRIESLK-LIYGIIGDEIAPTTGQRHLQGFLHLAGRGRTLEGLKKTLENNTVHLEPAKGSD-QQNKIYCSKE-NVLF---EHGVPTRPGSKRK-----LYERYEEDAEELRIEEPGAYRRCKMIE--KQKKWNYWVLNNPFPYDLYQWQEEL 163
BBTV_alphasat_Y_Rep_ACJ36781       ---MSS---FKWCFTLNYSSAAEREDFLSLLKEED-VHYAVVGDEVAPSTGQKHLQGYLSLK-KSIKLGGLKKRY-SSKAHWERARGTG-EDNSKYCSKE-TLIL---ELGFPVVNGSNKR----KISEMVARSPDRMKIEQPEMFHRYQSVN--CMKKFK---EEFVHPCLDRPWQIQL 157
BBTV_alphasat_HaiKou2_Rep_AEF97834 ---MSS---FKWCFTLNYSSAAEREDFLSRLKEDD-VYYAVVGDEVAPSSGQKHLQGYLSLK-KSMKLGGLKKRY-SSKAHWEKARGTD-KENSKYCSKE-TLIL---EIGFPATQGSNKR----KLSEMVSRSPERMRIEQPEIYHRYLSVS--KLKKFK---EEFVHPCLERPWQIQL 157
BBTV_alphasat_S1_Rep_AAG44003      ---MSS---FKWCFTLNYSSAAEREDFLALLKEEE-LNYAVVGDEVAPSSGQKHLQGYLSLK-KSIKLGGLKKKY-SSRAHWERARGSD-EDNAKYCSKE-TLIL---ELGFPASQGSNRR----KLSEMVSRSPERMRIEQPEIYHRYTSVK--KLKKFK---EEFVHPCLDRPWQIQL 157
CBDV_alphasat_Tung1_Rep_BAS02103   ---MAS---NKWCFTLNYSDAAERDIFLATLKEEE-LRYAVVGDEVAPTTGQKHLQGYLSLR-KLIRLGGLKKRF-SPKAHWEMAKGSD-EDNARYCSKE-TLIL---QLGIPTSQGSN-----------TRSPERMRIEQPEIFHRYESVK--KATIFK---DEYQHPSLDRQWQIQL 149
CBDV_alphasat_Fr_X7_Rep_YP_008854133 ---MAS---FKWCFTLNYSDAAERDIFLATLKKED-VHYAVVGDEVAPSTGQKHLQGYLSLK-RRIRLGGLKKKY-SPRAHWEVARGTD-GENMRYCSKE-TLIL---ELGLPATPGSNKR----KYAEMYTRSPERMKIEEPEIYHRYDSVN--CMKQFK---QEFVYPCLDRPWQIQL 157
BBTV_alphasat_4_Rep_ACB86656       ---MSA---SRWTFTLHYSDATERGKFLATLKEED-VHYAVVGDETAPNTGRKHLQGYLSLK-KRFRISGIKKKY-SSRAHWEKARGSD-YDNKAYCSKE-ALIL---ELGVPCQTGSNKR----KLADMVTRSPERMKIEQPEIFHRYASVK--KMKEFK---ERYVYPILDRPWQVQL 157
BBTV_alphasat_TW3S2_Rep_ACJ36782   ---MSS---FKWCFTLNYSSAAEREDFLALLKEED-VHYSVVGDEVAPATGQKHLQGYLSLK-KSIRLGGLKKKY-GSRAHWEIAKGTD-EQNRRYCSKE-TLVL---ELGTPVVPGSKKR----KLLDRFRESPEELKMDDPSKYRRCLAVE--SIKDAR---NNSEWVHELKEWQNKL 157
MDV_alphasat_C2_Rep_BAA33981       ---MAS---KRWCFTLNYKTALERETFISLFSRDE-LNYFVCGDEIAPTTGQKHLQGYVSMK-KLIRLGGLKKKF-GSIAHWEIAKGDD-FQNRDYCTKE-TLIA---EIGAPVKKGSNRR----KIMEIYEEDPEEMKLRDPDTALRCKAKK--LREEYC----SEVSVFSLRPWQIEL 156
FBNYV_alphasat_C1_Rep_CAA06791     ---MAS---KRWCFTLNYKTAVERESFISLFSRDE-LNYFVCGDETAPTTNQKHLQGYVSLK-KMIRLGGLKKKF-GYRAHWEIARGDD-FQNRDYCTKE-TLIS---EIGAPVKKGSNQR----KIMDLYLQDPEEMQLKDPDTALRCNAKR--LRIEYC----SSFAVISLRPWQSEL 156
SCSV_alphasat_C2_Rep_AAA68018      ----MA---RRYCFTLNYATEIERETFLSLFSQDE-LNYFVVGDETA-TTGQKHLQGFVSFK-NKIRLGGLKKKF-GNRAHWEIARGSD-SQNRDYCCKE-TLIS---EIGIPVMKGSNKR----KTMEIYEEDPEEMQLKDPDTALRCKAKK--LKEEYC----SCYDFQKLRPWQIEL 154
FBNSV_alphasat_1_Rep_AHC72219      ---MAC---ANWVFTRNFQGALP------SLSFDERVQYAVWQHERG---THDHIQGVIQLK-KKARFSTVKEII-GGNPHVEKMKGTI-EEASAYVQKEETRVAGPWSYGDLLKRGSHRR----KTMERYLEDPEEMKLKDPDVALRCNAKR--LKEDYC----SCFSSFKLRPWQIEL 152
BMLRV_alphasat_1_Rep_AHC72176      ---MAC---ANWVFTRNFQGALP------ILSFDERVQYAVWQHERG---THDHIQGVIQLN-KKARFSTVKEII-GGNPXLEKMKGTI-EEASAYVQKEKTRVAGPWSYGELVKKGSHKR----KTMERYLEDPEEMRLKDPDTALRCNAKR--LKEDYC----LSFACIQLRSWQIEL 152
FBNYV_alphasat_C7_EG93_Rep_CAB44025 ---MPSIRATHWCFTLNFS-GSIPE--IN---WTADVQYSIWQHERVG---HDHLQGYIQMNKHVT-LKKMKELLPG--AHLEMAKAP--KKAIEYCQKKESAIAGPWEYGTWISSGSHKR----KLMERFEDGPEEMKLEDPGLYRRCLSRV--QMKKIRES--CTWNFD-LRPWQDEL 154
MDV_alphasat_C10_Rep_BAA34048      ---MPSIRAIHWCFTLNFS-GKIPE--IV---WTADVQYSIWQHERVN---HDHLQGYIQMKKQTT-LKKMKELLPG--AHLEMARAP--KKAIDYCQKKETAIDGPWEYGTWISTGSHKR----KLMERFDEDPEEMKLEDPGLYRRCLSRV--QMTKVREK--NSWDYD-LRPWQDEL 154
MDV_alphasat_C3_Rep_BAA33982       ---MPTVQSTCWVFTLNFK-GEIPI--LP---FNERVQYACWQHERVG---HDHLQGFIQMKAQQS-LGQMKAIIPG--AHFEKMRALNSDQAKAYAMKEDTRIEGPWEYGKYIKKGSHKR----KIMERFEDDPEEMKIEDPSLYRRCLSRK--MTEEQRST--AEWNYD-MKPWQDQV 156
PNYDV_alphasat_3_Rep_AHC72280      ---MPSQKSTYWVFTLNFK-GEIPI--LS---LDTRVQYALWQHEYVS---HHHLQGFIQMKAQST-LGQMKALIPG--AHFEVMKALDSDQARSYAMKEDSRLEGPWEYGLYIKKGSHKR----KVMERFDSEPEEMKVEDPSLYRRCLSRK--MTEEQRSS--STWDYD-LRPWQDSI 156
SCSV_alphasat_C6_Rep_AAA68022      ---MPTRQSTSWVFTLNFE-GEIPI--LP---FNESVQYACWQHERVG---HDHLQGFIQFKSRNTTLRQAKYIFNG--LNPHLEIARDVEKAQLYAMKEDSRVAGPWEYGLFIKRGSHKR----KLMERFEEDGEEMKIADPSLYRRCLSRK--MAEEQRCS--SEWNYD-LRPWQEEV 157
BMLRV_alphasat_2_Rep_AHC72185      ---MPTLQGTFWCFTLNFS-GDVPA--LS---FDETVQYAVWQHECVS---HDHLQGFVQMKKRST-MKMMKILLPG--AHLEVAKGT-PEQASSYAMKESSRVAGPWFYGELLKKGSNKR----KLLDRYRDSPEDMELEDPAKARRCRARI--DKEKFVSD--FKVEDD-EQEWKRLV 155
MDV_alphasat_C1_Rep_BAA33980       ---MPTLQGTFWCFTLNFS-GDAPS--LS---FNERVQYACWQHERVS---HDHLQGYIQMKKRST-LKMMKELLPG--AHLEVSKGT-PEEASDYAMKEETRVAGPWTYGELLKKGSNKR----KLLDRYKENPEDMELEDPAKARRCRAKI--DKEKFIAE--FKVEDD-EQEWKKIL 155
FBNSV_alphasat_2_Rep_AHC72218      ------MQSVSWVFTLNFS-GEVPL--LS---FDERVQYAVWQHERVN---HDHLQGVIQMKRKTR-MNTVKSLIGGN-PHLEPMRSR-IEEASAYAMKEESRVAGPWFHGELLKKGSNKR----SLMEKYKEDPVSMELEDPGKARRCRARL--DKEKFVAA--FKVEDD-EQEWKRLL 153
PNYDV_alphasat_1_Rep_AHC72279      ------MASCNWVFTRNFS-GDTPL--LS---FDERVQYACWQHEKVA---HDHLQGVIQLKKRAR-MNTVKALIGGN-PHVEMMKAK-IEEASAYVQKEESRVAGPWIYGEMLKKGSNKR----SLMERYKEDPELMELEDPGKARRCRARI--DKEKFVSE--FKVEDD-EQEWKSLL 153
PaLCuV_IN__alphasat_Rep_AFG29502   ---MPAVQSFWWCFTIFFTSAAPPD--LVSLFENTSVSYACWQEEESPTTKRRHLQGYLQLKTKKS-LSTVKSLFGDLNPHLEKQRARKTDDARDYCMKEETRVSGPFEFGDYCPSGSHKR----RQRESVIRSPVRMAEENPSVFRRVKAKI--AEEEFQKTA-HEIQISNLKSWQLRL 167
CLCuV_alphasat_Rep_AIH07616        ---MPAAQSFWWCFTIFFTTAAPPD--LVPLFENTSVSYACWQEEESPTTKRRHLQGYLQLKTKKS-LSAVKSLFGDLNPHLEKQRARRTDEARDYCMKEETRVSGPFEFGDYCPSGPHKR----RQRESVIRSPVRMAEENPSVFRRVKAKI--AEEEFQKTA-HEIQISNLKSWQLRL 167
GDS_alphasat_Rep_ALB26259          ---MPSAQSVFWCFTIFFTSASPPD--LVPLFENTSVSYACWQEEESPTTRRRHLQGYLQLKTKKS-LSAVKSLFGDLNPHLEKQRARRTDEARDYCMKEETRVSGPFEFGDYCPSGSHKR----RQRESVIRSPVRMAEENPSLFRRVKAKI--AEEEFQKTA-REIQILNLKSWQSRL 167
SiLCuV_alphasat_Rep_CBY89002       ---MPTIQSQWWCFTVFFLSAAAPD--LVPLFENTHVSYACWQEEESPTTRRRHLQGYLQLKGKRT-LAQVKSLFGDLNPHLEKQRARKTDEARDYCMKEETRVSGPFEFGDYCPSGSHKR----RQRESVIRSPVRMAEENPSLFRRVKAKI--AEDFQKTA-REIEILNLKSWQLRL 167
CLCuDaV_alphasat_Rep_CAD56270      ---MPALKAQWWCFTIFFVSAAAPD--LVPLFENTHVSYACWQEEESPTTRRRHLQGYLQLKGQRT-LNQVKVIFGELKPHLEKQRARRTDEARDYCMKEETRVSGPFEFGDYCPGGSHKR----RLRESVIRSPVRMAEENPSVFRRVKAKL--AEEEFQKSA-HEIQISNLKSWQTRL 167
ToLCV_alphasat_Rep_CDH98084        ---MAAIKSNWWCFTVFFLSATAPD--LVPLFENTHVSYACWQEEESPTTRRRHLQGYLQLKGQRT-LNQVKSIFGDLKPHLEKQRARKTDDARDYCMKPETRVSGPFEFGEYCPAGSHKR----RQRELVIRSPVRMAEENPSVFRRVKAKI--AEEEFQKSA-PEIQISNLKSWQSRL 167
TbCSV_alphasat_Rep_CBK44088        ---MAALKAQWWCFTVFFLSATAPD--LVPLFENTHVSYACWQEEESPTTRRRHLQGYLQLKGQRT-LNQVKAIFGDLKPHLEKQRARKTDEACDYCMKEETRVSGPFEFGEYCPAGSHKR----RQRELVIRSPVRMAEENPSVFRRVKAKI--AEEEFQKSA-HEIQISDLKSWQLRL 167
AEV-IN_alphasat_Rep_CEE15328       ---MAALKGQWWCFTVFFLTATAPD--LVPLFENTHVSYACWQEEESPTTKRRHLQGYLQLKGQRT-LNQVKAIFGDLKPHLEKQRARKTDEARDYCMKEETRVSGPFEFGEYCPAGSHKR----RQREIVNRSPVRMAEENPSVFRRVKAKI--AEEEFQKSA-HEIQISNLKSWQLRL 167
AYVV_alphasat_Rep_CAB59911         ---MPALKGQWWCFTVFFLSSTAPD--LVPLFENTHVSYACWQEEEAPTSKRRHLQGYLQLKGQRT-LNQVKALFGELKPHLEKQRARKSDEARDYCMKEETRVSGPYEFGDYCPSGSHRR----RQRELVIRSPVRMAEENPSVFRRVKAKI--AEEEFQATV-AEIQISNLKSWQLRL 167
TbCSV_alphasat_Y290_Rep_CAI59924   ---MPSITSVFWCFTIFFTSASAPD--LVPLFENTHVSYACWQEEESPTTRRRHLQGYLQLKGKRT-LNQVKAIFGDLKPHLEKQRARKTDEACDYCMKEETRVSGPFEFGDYCPSGSHKR----RQRESVIRSPVRMAEENPSVFRRVKAKL--AEEEFQATV-AEIQISNLKSWQLRL 167
AYVIV_alphasat_Rep_CDH98093        ---MPTIQSQWWCFTVFFLSATAPD--LVPLFENTHVSYACWQEEESPTTRRRHLQGYLQLKGKRS-LAQVKALFGDLNPHLEKQRARKTDDARDYCMKEETRVTGPFEFGEYCPAGSHKR----RQRESVIRSPVRMAEENPSVFRRVKAKI--AEEEFQKSA-SGIQISNLKSWQLRL 167
CYMV_alphasat_Rep_CBJ19303         ---MPTIQSQWWCFTVFYLSATAPD--LVPLFENTHVSYACWQEEESPTTGRRHLQGYLQLKGKRS-FAQVKALFGDLNPHLEKQRARKTDEARDYCMKEETRVSGPFEFGDYCPSGSHRR----RQRESVIRSPVRMAEENPSVFRRVKAKI--AEDFQKTA-KEIQISNLKSWQLRL 167
VEM_alphasat_2_Rep_ALK03648        ---MKTPQSIWWCFTIFFASAGAPE--LFPLFENTSVSYACWQEEESPTTHRRHLQGYLQLKSKKT-LNQVKALFGDLNPHLEKQRARNSDDARDYCLKEASRVSGPWEFGEYCPSGSHKR----RQREQVQKSPERMMEENPSVYRRVKARL--SIEAHQLSL-SELPFL-LKPWQFEI 166
Dragonfly_ass_alphasat_Rep_AFV91336 MGSFSSPRSRWWCFTLNFS-GDPPE--LAWWLARDEIKYACWQHEKG---THDHMQGYLQLKKPSR-LTAVRKLFGNFRPHLEVQKARRAEDARDYCMKDESRVAGPWEIGEFIPQGSHKG----RMRDLVRRSPERMAEENPSVYRRVLAAQ--SVERFRSDP-SLLPDP-LRDWQICL 165
EuYMV_alphasat_Rep_CBA18108        ---MSGR-SRNWCFTL-FEYTLPL-----FLSLPDWAVYLVFQEEVSPSTTRKHIQGYCTLK-KPQRLSFLKTKLG-NGAHLEIAKGSA-SSNRDYCRKDDSRASGPWEFGILAEQGSHKR----KTMERFQEDPDEVRLADPKLYRRCLATK--INKEFS----CLVLPAFDRPWQLLV 157
CLCuGeV_alphasat_Rep_CDI44972      ---MAPQ-GKRWCFTS-FEEREP------FTSLPDGCSYVVXQREKCPETGREHWQGFVTFS-AARRLNSVKSFAGGNNWHWEVARGTN-SECRSYCTKEDTRVAPPVEYGVFCELGSKKR----KVMEQFEEDPEELKLADPSKYRRCLAIV--TNKRFN----NVELPLFDRPWQEEL 157
SCSV-[AU;F]_M_Rep_CAB96405         ---MAR-QVICWCFTLNNP---L-----APLSLHESMKYLVYQTEAGDN-GTIHYQGYVEMK-KRTSLVQMKKLLPG--AHLEKRRGSQ-GEARAYAMKEDSRVEGPWEFGEFKEVLEDKLRSVMEDMKSTGKRPVEYIEDCCNTYDKSSATLREFRGELKKKQAIEEWQLQRQPWMDEV 163
FBNYV-[EG;1_93]_M_Rep_NP_619567    ---MAR-QVICWCFTLNNP---L-----SPLSLHDSMKYLVYQTEQGEA-GNIHFQGYIEMK-KRTSLAGMKKLIPG--AHFEKRRGTQ-GEARAYSMKEDPRLEGPWEYEEFVPTIEDKLREVMNDMKITGKRPIEYIEECCNTYDKSASTLREFRGELKKKKAISSWELQRKPWMDEV 163
MDV-[JP;1]_M_Rep_NP_619769         ---MAR-QVICWCFTLNNP---L-----SPLSLHELMKYLVYQREQGEA-GNIHFQGYIEMK-KRTSLAGMKKLIPG--AHFEKRRGTQ-GEARAYAMKEDTRLEGPWEYGEFIPTIEDKLREVMNDMKITGKRPIEYIEECCNTYDKSASTLREFRGELKKKKAIISWELQRKPWMDEV 163
FBYLV-[ET;231]_M_Rep_CCF74113      ---MAR-QVICWCFTLNNP---L-----SPLSLHESMKYLVYQTEQGDS-GNIHFQGYIEMK-KRTSLAGMKKLIPG--AHFEKKKGTQ-GQARAYAMKEDTRVEGPWEYGEFIPTIEDKLRDVMQDMKNTGKRPIEYIEECCDTYDKSASTLREYRGELKKKQAIASWELQRKPWMDEV 163
FBNSV-[ET;Hol_97]_M_Rep_ADJ00306   ---MAR-QVICWCFTLNNP---L-----SPIFLHESMKYLVYQTEQGES-GNIHFQGYIEMK-KRTSLAGMKRLIPG--AHFEKRRGTQ-GEARAYAMKEESRIEGPWEFGEFIASVEDKLREVMNDMKNTGKRPIEYIEECCNTYDKSASTLREFRGELKKKKAIATWELQRKPWMDEV 163
PNYDV-[DE;15]_M_Rep_ADE87486       ---MSR-QVICWCFTLNNP---L-----SLLSLHESMKYLVYQREQGES-GNIHFQGYVEMK-KRTSLAGMKRLIPG--AHFEKRRGTQ-GEARAYAMKEDSRLEGPWEEGEFHLTVEDKLREVMEDMKNTGKRPIEYIEECCNTYDKSSGTLREYQGELRKKRAINGWQLQRKPWMDEV 163
PYSV-[AT;15]_M_Rep_AHC72281        ---MSR-QVICWCFTLNNP---L-----SPLSLHESMKYLVYQREQGES-GNIHFQGYIEMK-KRTSLAGMKRLIPG--AHFEKRRGTQ-GEARAYAMKESSRLEGPWEEGEFHLTVEDKLREVMEDMKNTGKRPIEYIEECCNTYDKSSGTLREYQGELRKKRAISEWELQRKPWMDEV 163
BBTV-[Au]_M_Rep_AAB25544           ---MAR-YVVCWMFTINNP---T-----TLPVMRDEIKYMVYQVERGQE-GTRHVQGYVEMK-RRSSLKQMRGFFPG--AHLEKRKGSQ-EEARSYCMKEDTRIEGPFEFGSFKLSCNDNLFDVIQDMRETHKRPLEYLYDCPNTFDRSKDTLYRVQAEMNKTKAMNSWRTSFSAWTSEV 163
BBTV-[TW]_M_Rep_ABH03036           ---MAR-YVVCWMFTINNP---A-----SLPVMRDEFKYMVYQVERGQE-GTRHVQGYVEMK-RRSSLKQMRGFFPG--AHLEKRKGSQ-EEARAYCMKEDTRIEGPFEFGAFKLSCNDNLFDVIQDMRETHKRPLEYLYECPNTFDRSKDTLYRVQAELNKTKAMNSWKTSFNAWTSEV 163
CBDV-[IN]_M_Rep_AGG38934           ---MAR-YVVCWMFTINNP---S-----SLPVMRDEIKYMVYQVERGQE-GTRHVQGYVEMK-RRSSLKQMRMFFPG--AHLEKRKGTQ-EEARTYCMKEDTRIEGPFEFGVFKLACNDSLYDVIQDMRLTNKRPLEYLYDCPNTFDRSKDTLYRVQAEMNKTKAMESWGKSYGSWTEQV 163
ABTV-[MY]_M_Rep_ABP96965           ---MAR-YVVCWMFTINNP---E-----ALPEMREEYKYLVYQVERGES-GTRHVQGYVEMK-RRSSLKQMRALIPG--AHLEKRRGTQ-EEARAYCMKADTRVEGPFEFGLFKVSCNDNLFDVIQDMRETHKRPIEYLYDCPNTFDRSKDTLYRVQAEMNKMQAMMSWSETYGCWTKEV 163
ABTV-[PH]_M_Rep_ABP96960           ---MAR-YVICWMFTINNP---E-----TLPPMRDEYKYMVYQVERGES-GTRHVQGYVEMK-RRTSLKQMRALIPG--AHLEKRKGTQ-EEARSYCMKADTRIEGPFEYGMFKISCNDNLYDVIQDMRETNKRPIEYLYDCPNTFDRSRDTLYRVQAELNKMQAMKSWSESYGCWTKEV 163
```

A

```
                                          EELLEEEPDDRTIIWVYGPXGGEGKSTFAKHL-IKRDWFYTRGGKTDDVLYQYIGE-PGRNVVFDIPRCK-EDYLNYSLIEMLKNRXIVSXKYEPVMXPLINX--VHVIVMANFLPDF-------------GKISEDRIKIIYC----------------------
                                                   190       200       210       220       230       240       250       260       270       280       290       300       310       320       330       340
CFDV-[VU;89]_alphasat_R_Rep               LSAIEQPADDRTIFWVCGREGGDGKSVFAKYLGLKPDWFYTCGGTRKDVLYQYIED-PKRNLILDVPRCN-LDYLNYALLECVKNRAFSSDKYEPLSYLGFDH--VHVIVFANVLPDY-------------LKISRDRIKLWNV          290
CFDV-[VU;89]_alphasat_1_Rep               LSAIGEPADDRTILWICGRDGGDGKSVFAKYLGLKPDWFYTCGGTRKDVLYQYIED-PKRNLILDVPRCN-LEYLNYALLECVKNRAFSSDKYEPLSYLGFDH--VHVLVFANVLPDY-------------LKISRDRIKLWNI          290
CFDV-[VU;89]_alphasat_2_Rep               LSAIDQPADDRTIIWICGRDGGEGKSVFAKYLGLKPDWFYTCGGTRKDVLYQYIED-PKRHMILDVPRCS-VEHLNYALLECVKNRAFSSDKYEPLSYLGFDH--VHVLVFANVLPDY-------------MKISEDRIKLFDI          289
CFDV-[VU;89]_alphasat_4_Rep               LSEIQEQADDRTILWVCGRTGGEGKSVFAKFLGLKPDWFYTCGGTRKDVLYQYIEE-PKRNVILDVPRCN-LEHLNYALLECIKNRAFSSDKYEPISYVGFDN--VHVIVFANILPDY-------------LKISRDRIKLWDI          290
CFDV-[VU;15]_alphasat_8_Rep               LSQIDGQADDRTILWVCGRTGGEGKSVFAKYLGLKPDWFYTCGGTRKDVLYQYIED-PKRNVILDVPRCN-LEHLNYALLECIKNRAFSSDKYEPLSYLGFDN--VHVIVFANVLPDY-------------LKISRDRIKLWDI          290
CFDV-[VU;89]_alphasat_5_Rep               LAAIEEPPDDRTIIWVCGRSGGEGKSVFGKFLGLKPDWFYTCGGSKKDILYQYIEN-PKRHVIMDFPRCT-DTYMNYAVLECMKNRAFSSDKYEPLSYLGFDN--IHVIVLSNVLPDY-------------FKISEDRIKLINI          290
CFDV-[VU;89]_alphasat_3_Rep               REAISEAPDDRSIIWVYGPDGNEGKSTFAKSL-IKQDWFYTRGGKKENILFSYIDEGSEKNIVFDIPRCN-QDYLNYDVIEALKDRVIESTKYKPVKIIELCN--IHVVVMANFLPDY-------------MKISEDRIKIIRCY         287
CFDV-[VU;89]_alphasat_6_Rep               GEAISEAPDDRSIIWVYGPDGNEGKSTFAKSL-IKQDWFYTRGGKKENILFSYIDEGSEKNIVFDIPRCN-QDYLNYDVIEALKDRVIESTKYKPVKIIELCN--IHVVVMANFLPDY-------------MKISEDRIKIIRCY         287
CFDV-[VU;89]_alphasat_7_Rep               MEVLQEPADNRTILWICGRDGGDGKTEFGKYLGLNEKWFYSCGGKTRDVLYQYIEE-PERNFILDVPRCN-DQYMNYALIECIKNRAFSSDKYEPLQYLGFDK--VHVIVLCNFLPDY-------------LKISEDRIKLWNI          290
BBTV_alphasat_Y_Rep_ACJ36781              TEAIDEEPDDRSIIWVYGPYGNEGKSTYAKSL-IKKDWFYTRGGKKENILFSYVDEGSDKHIVFDIPRCN-QDYLNYDVIEALKDRVIESTKYKPIKIVELGK--IHVIVMANFMPDF-------------CKISEDRIKIIYC          284
BBTV_alphasat_HaiKou2_Rep_AEF97834        TEAIAEEPDDRSIIWVYGPNGNEGKSTYAKSL-IKKDWFYTRGGKKENILFSYVDEGSTKHIVFDIPRCN-QDYLNYDVIEALKDRVIESTKYKPIKIVELDR--IHVIVMANFMPDF-------------CKISEDRINIIYC          284
BBTV_alphasat_S1_Rep_AAG44003             TEAIDEEPDDRSIIWVYGPNGNEGKSTYAKSL-MKKDWFYTRGGKKENILFSYVDEGSEKHIVFDIPRCN-QDYLNYDVIEALKDRVIESTKYKPIKLVELIN--IHVIVMANFMPEF-------------CKISEDRIKIIYC          284
CBDV_alphasat_Tung1_Rep_BAS02103          TEAIAEEADDRTIIWVYGPNGNEGKSTYAKSL-MKKDWFYTRGGKKENILFSYVDEGSTKHVVFDIPRCH-QEYLNYDVIEAIKDRVIESTKYKPIKIIEVNR--VHVIVMANFMPDY-------------CKISEDRIKTINC          276
CBDV_alphasat_Fr_X7_Rep_YP_008854133      TEAINEEPDDRSIIWVYGSNGNEGKSTYAKSL-IKKDWFYTRGGKKENTLFSYIDEGDEKNVVFDIPRCN-QDYLNYDVIEALKDRVIESTKYKPVKKVRMNR--IHVIVMANFMPDY-------------CKISEDRIKIIYC          284
BBTV_alphasat_4_Rep_ACB86656              TELIEAEPDDRTIIWVFGPKGNEGKSTYAKSL-IQKDWFYTRGGKKENILFAYVDEGSTKNVVFDLPRTV-QEFINYDVIEALKDRVIESTKYKPVKYLELNT--VHVLVMANFLPDM-------------CKISEDRIKIVAC          284
BBTV_alphasat_TW3S2_Rep_ACJ36782          IQHIEGVPDDRSIIWVYGPNGGEGKSTFARYLSLKPGWGYINGGKTSDMMH-IITMDPDNNWIIDIPRSN-SEYLNYGVIEQIKNRVLINTKYEPCVIRKDGQN-VHVIVLANVLPDY-------------CKISEDRIKIINC          285
MDV_alphasat_C2_Rep_BAA33981              HRALMEVPDDRTIIWAYGPDGGEGKSTFAKEL-IKYGWFYTAGGKTQDILYMYAQD-PERNIAFDVPRCS-SEMMNYQAMEMMKNRVFASTKYRPVDLCIRKK--VHLIVFANVAPDP-------------TKLSEDRIVIINC          282
FBNYV_alphasat_C1_Rep_CAA06791            HRVLMAEPDDRTIIWVYGSDGGEGKSTFAKEL-IKYGWFYTAGGKTQDILYMYAQD-PERNIAFDVPRCS-SEMMNYQAMEMLKNRVFASTKYRPVDLCVRKK--VHLIVFANVSPDP-------------TKISEDRIVIINC          282
SCSV_alphasat_C2_Rep_AAA68018             HAALMAEPDDRSIIWVYGSDGGEGKTSFAKEL-IRYGWFYTAGGKTQDVLYMYAQD-PERNIAFDVPRCS-SEMMNYQAMEMLKNRVFASTKYRPVDLCIRKL--VHLIVFANVAPDP-------------TRISEDRLVIINC          280
FBNSV_alphasat_1_Rep_AHC72219             HRVLMNEPDDRSIIWVYGPDGGEGKSTFAKEL-IKYGWFYTAGGKTQDVLYMYAQD-PERNIAFDVPRCS-SEMMNYQAMEMMKNRCFASTKYRSIDLCVRKN--VFLVVFANVEPDP-------------TKISGDRIVIINC          278
BMLRV_alphasat_1_Rep_AHC72176             HTALMEEPEDRTIIWVVGSDGGEGKSTFAKEL-IKYGWFYTAGGKTQDILYMYAQD-PERNIAFDVPRCS-SDMMNYQAMEMMKNRCFASTKYRSVDLCVKKK--IHLVVFANVSPDP-------------TKISEDRIVIINC          278
FBNYV_alphasat_C7_EG93_Rep_CAB44025       LKTIEQEPDDRTIIWVYGPHGGEGKSAFAKYLTLKEGWWYTAGGKATDMLYSYSLD-PTCHVCIDIPRCTREEYINYSVIEQIKNRVIINTKYEPCTIRDDGHN-VHVIVFCNFLPDV-------------TRISEDRIKIINC          283
MDV_alphasat_C10_Rep_BAA34048             LKTIEQEPDDRTILWVYGPHGGEGKSVFAKYLTLKEGWWYTAGGKATDMLYSYSLD-PTCHVCIDIPRCTKEEYINYAVIEQIKNRIIINTKYEPCTIRDDGHN-VHVIVFANYLPDV-------------TRISEDRIKIIYC          283
MDV_alphasat_C3_Rep_BAA33982              IQEIEETPDYRKIIWVYGPKGGEGKSTFARYLSLKQGWGYLPGGPTHDMLHIISGE-PKNNWVFDIPRVA-SEYVNYGVLEQVKNRVMVNTKYEPIVIRDDNHP-VHVIVFANCMPDF-------------TKISEDRMKIVHC          284
PNYDV_alphasat_3_Rep_AHC72280             LEAIQLPPNYRDVLWVYGPNGGEGKSTFARHLSLKDGWGYLPGGKTQDMMHLITAE-PKNNWVFDIPRVS-SDYINYGVIEQVKNRVMVNTKYEPVIMRDDNNP-VHVIVFANCLPDV-------------TKLSEDRIKMIYC          284
SCSV_alphasat_C6_Rep_AAA68022             MHLLEEEPDYRTIIWVYGPAGNEGKSTFARHLSLKDGWGYLPGGKTQDMMHLVTAE-PKNNWVFDIPRVS-SEYVNYGVIEQVKNRVMVNTKYEPCVMRDDNHP-VHVIVFANVLPDL-------------GKLSEDRIKLIRC          285
BMLRV_alphasat_2_Rep_AHC72185             EEELNIEASPRSIIWVYGPQGGEGKTTKAKEL-ITRGWFYTRGGKKDDVAYSYVED-PTRHVVFDIPRDM-QDYCNYSLIEMLKDRIIISNKYEPITNCQVAN--IHVIVMANFLPDV-------------TKISNDRLKIIYC          281
MDV_alphasat_C1_Rep_BAA33980              EKEIEKIASPRSILWVYGPQGGEGKTSKAKEL-ITRGWFYTRGGKKDDVAYSYVED-PTRHVVFDIPRDM-QEYCNYSLIEMLKDRIIISNKYEPITNCQVYN--IHVIVMANFLPDV-------------TKISEDRIKIIYC          281
FBNSV_alphasat_2_Rep_AHC72218             EEELEKVPSPRSILWVYGPTGGEGKTTKAKEL-ITRGWFYTRGGRKDDVAYSYIED-PTRHVVFDIPRDM-KDYCNYSLIEMLKDRIIISNKYEPVTNCQLSN--IHVIVMANFLPDY-------------CKISEDRINIIYC          279
PNYDV_alphasat_1_Rep_AHC72279             EEDLKKEASPRSIMWVYGPHGGEGKTTKAKEL-ITRGFFYTRGGKKDDVAYSYIED-PTRHVVFDIPRDM-QEYCNYSLIEMLKDRIIISNKYEPITNCQLDN--IHVIVMANFLPDV-------------TKISNDRIKIIYC          279
PaLCuV-IN__alphasat_Rep_AFG29502          KTLLERAPDDRTIFWVYGPDGGEGKSTFARDLYRSGTWFYTRGGSADNVAYQYIGQ-LGNNIVFDIPRDK-KDYLQYSLIEMFKDRLIVSNKYEPLMAPLINC--IHVVVMSNFLPDF-------------EKISQDRVHVIPCIPCGVCRKQHHVGEKCDEYVE          315
CLCuV_alphasat_Rep_AIH07616               KTLLDRDPDDRTIFWVYGPDGGEGKSTFARDLYRSGTWFYTRGGSADNVSYQYIGQ-LGNNIVFDIPRDK-KDYLQYSLIEMFKDRLIVSNKYEPLMAPLINC--IHVVVMSNFLPDF-------------EKISVDRVHVIPCRPCGVCLKHHNINDNCEDYID          315
GDS_alphasat_Rep_ALB26259                 QTLLERDPDDRTIFWVYGPTGGEGKSTFARDLYRSGTWFYTRGGSADNVSYQYIGQ-LGNNIVFDIPRDK-KDYLQYSLIEMFKDRLIVSNKYEPIMAPLINC--IHVVVMSNFMPDF-------------EKISQDRVHVIPCRPCGVCLKHHIIEMKCEEYVD          315
SiLCuV_alphasat_Rep_CBY89002              QTLLERAPDDRTIFWVYGPTGGEGKSTFARDLYRSGTWFYTRGGSADNVSYQYIGQ-LGNNIVFDIPRDK-KDYLQYSLIEMFKDRLIVSNKYEPLMAPLINC--IHVVVMSNFMPDF-------------EKISQDRVHVIPCRPCGVCLKHHIIEMKCEEYVD          315
CLCuDaV_alphasat_Rep_CAD56270             KTLLERAPDDRTIFWVYGPNGGEGKSTFARDLYRSGTWFYTRGGSADNVAYQYIGC-LGNNIVFDIPRDK-KDYLQYSLIEMFKDRLIVSNKYEPLMAPLINC--IHVVVMSNFMPDF-------------EKISQDRVHVIPCRPCGVCLKHHIIEMKCDDYMD          315
ToLCV_alphasat_Rep_CDH98084               KTLLDRDPDDRTIFWVYGPYGGEGKSTFARDLYRSGSWFYTRGGSADNVAYQYIGC-LGNNIVFDIPRDK-KDYLQYSLIEMFKDRLVVSNKYEPLMAPLLKC--IHVVVMSNFLPDF-------------EKISVDRVHVIPCIPCGVCLKHHVDDVMCGEYME          315
TbCSV_alphasat_Rep_CBK44088               KTLLDRDPDDRTIIWVYGPTGGEGKSTFARDLYKSGSWFYTRGGSADNVSYQYIGQ-LGNNIVFDIPRDK-KDYLQYNLIEMFKDRLIVSNKYEPLMAPLINC--IHVVVMSNFLPDF-------------EKISSDRVHVIPCIPCGVCLKHHIDEIRCSEYFD          315
AEV-IN_alphasat_Rep_CEE15328              KTLLDRDPDDRTIIWVYGPIGGEGKSTFARDLYRSGSWFYTRGGSADNVSYQYIGQ-LGNNIVFDIPRDK-KDYLQYSLIEMFKDRLIVSNKYEPLMAPLINC--IHVVVMSNFLPDF-------------EKISNDRVHVIPCIPCGVCLKHHIHEIKCDEYLD          315
AYVV_alphasat_Rep_CAB59911                KTLLDRDPDDRTIFWVYGPIGGEGKSTFARDLYRSGSWFYTRGGSADNVSYQYIGC-LGANIVFDIPRDK-KDYLQYSLIEMFKDRLIVSNKYEPLMAPLINC--IHVVVMSNFLPDF-------------DKISEDRVHVIPCIPCGACLKHHIGLIKCDEYLD          315
TbCSV_alphasat_Y290_Rep_CAI59924          RTLLDRDPDDRTILWVYGPTGGEGKSTFARDLYRSGSWFYTRGGSADNVSYQYIGC-LGNNIVFDIPRDK-KDYLQYSLIEMFKDRLIVSNKYEPLMAPLVNC--IHVVVMSNFLPDF-------------EKISSDRVHVIPCIPCGVCLKHHIGEIKCDEYLD          315
AYVIV_alphasat_Rep_CDH98093               KTLLDRDPDDRTIIWVYGPTGGEGKSTFARDLYRSGSWFYTRGGSADNVAYQYIGC-LGNNIVFDIPRDK-KDYLQYSLIEMFKDRLIVSNKYEPLMAPLINC--IHVVVMSNFLPDF-------------EKISSDRVHVIPCIPCGVCLKHHIHEIKCDEYLD          315
CYMV_alphasat_Rep_CBJ19303                KTLLDRDPDDRTIIWVYGPTGGEGKSTFARDLYRSGSWFYTRGGSADNVSYQYIGC-LGHNIVFDIPRDK-KDYLQYSLIEMFKDRLIVSNKYEPLMAPLINC--IHVVVMSNFLPDF-------------EKISMDRVHVIPCIPCGVCLK                      302
VEM_alphasat_2_Rep_ALK03648               ERVISRPASTRDIIWVYGSTGGEGKSTYARELLRRG-WFYTRGGSVDNVAYQYLDN-VDRDVVFDIPRDK-KDYLQYSLIEMIKDGVIISNKYEPVTAVCSRP--RHVVVMSNFLPDF-------------SKISVDRVSVIPCIPCGLCLNCHSSDVFCLEFNNE         314
Dragonfly_ass_alphasat_Rep_AFV91336       LELLEEVPDNRSIIWIYGPAGAEGKTMMAKELFRRG-WFYTSGGTADNVKYQYAVE-VESHVVFDIPRDK-KDYIQYSLIEELKNGMIVSNKYEPIRVVRISGREVHVVVMCNFMPDY-------------SKISPDRIVIVYCRKCPSCETCLCIGC                307
EuYMV_alphasat_Rep_CBA18108               EKVLDEGPDDRTIIWVYGSQGNEGKTTWAKSK-IQSGWFYSRGGKGENIKYSYAEH--LGHCIFDLPRQV-EDVLQYTVLEEIKDRLIRSSKYEPIDFNCSDK--VHVVVLSNFLPQIDLEYDSKGNLVKKQLLSKDRLCIINIDESVIVRNDQTVTFDEYIE           314
CLCuGeV_alphasat_Rep_CDI44972             TSLLNLEPDDRTIIWVYGSQGNEGKTTYVKSL-IQKGWFNSRGGKSDDIMFQYIIH--GGNAVFDFPRDK-EDYVNYNVLEAIKDRTCISNKYEPLSINYPNL--VHVVVMANFKPQLEDEFDDKGRIVKKQMLSVDRIKLIEC                          295
SCSV-[AU;F]_M_Rep_CAB96405                ERLMET-KDCRRIIWVYGPQGGEGKTSYAKHLVKTRDAFYSTGGKTADIAFAWDHQ---ELVLFDFPRSF-EEYVNYGVIEQLKNGIVQSGKYQSIVKYCNY---VEVIVFANFIPRS-------------GMFSEDRIVIVYA          286
FBNYV-[EG;1_93]_M_Rep_NP_619567           DALLQE-RDGRRIIWVYGPQGGEGKTSYAKHLVKTRDAFYSTGGKTADIAFAWDHQ---ELVLFDFPRSF-EEYVNYGVIEQLKNGIIQSGKYQSVIKYSDY---VEVIVFANFTPRS-------------GMFSEDRIVYVYA          286
MDV-[JP;1]_M_Rep_NP_619769                DTLLQE-RDGRRIIWVYGPQGGEGKTSYAKHLVKTRDAFYSTGGKTADIAFAWDHQ---ELVLFDFPRSF-EEYVNYGVIEQLKNGIIQSGKYQSVIKYSDY---VEVIVFANFTPRS-------------GMFSDDRIVYVYA          286
FBYLV-[ET;231]_M_Rep_CCF74113             DAMLEE-RDGRRIIWVYGPLGGEGKTSYAKHLVKTRDAFYSTGGNTADIAFAWDHQ---ELVLFDFPRSF-EEYVNYGAIEQLKNGIIQSGKYQSVIKYTDY---VEVIVFANFTPRS-------------GMFSDDRIVFVYA          286
FBNSV-[ET;Hol_97]_M_Rep_ADJ00306          DALLQE-RDGRRIIWVYGPQGGEGKTSYAKHLVKTRDAFYSTGGKTADIAFAWDHQ---ELVLFDFPRSF-EEYVNYGVIEQLKNGIIQSGKYQSVIKYSDY---VEVIVFANFTPRS-------------GMFSDDRIVFVYA          286
PNYDV-[DE;15]_M_Rep_ADE87486              ESLLET-RDGRRIIWVYGPQGGEGKTSFAKHLVKTRDAFYSTGGKTADIAFAWDHQ---PIVLFDFPRSF-EEYVNYGVMEQLKNGIIQSGKYQSVIKYAEY---VEVIAFANFTPRS-------------GMFSEDRIIIVNV          286
PYSV-[AT;15]_M_Rep_AHC72281               ERLMET-KDCRRIIWVYGPQGGEGKTSFAKHLVKTRDAFYTTGGKTADIAFAWDHQ---PIVLFDYPRSF-EEYVNYGVMEQLKNGIIQSGKYQSVIKYCNY---VEVIAFANFTPRS-------------GMFSEDRIIIVNA          286
BBTV-[Au]_M_Rep_AAB25544                  ENIMAQ-PCHRRIIWVYGPNGGEGKTTYAKHLMKTRNAFYSPGGKSLDICRLYNYE---DIVIFDIPRCK-EDYLNYGLLEEFKNGIIQSGKYEPVLKIVEY---VEVIVMANFLPKE-------------GIFSEDRIKLVSC          286
BBTV-[TW]_M_Rep_ABH03036                  ENIMAE-PCYRRIIWVYGPNGGEGKTTFAKHLMKTKNAFYSPGGKSLDICRLYNYE---DIVIFDIPRCK-EEYLNYGLLEEFKNGIIQSGKYEPVLKIVEY---VEVIVMANFLPKE-------------GIFSEDRIKLVAC          286
CBDV-[IN]_M_Rep_AGG38934                  EELMAQ-PCHRRIIWVYGPNGGEGKTTYAKHLMKTRNAFYTPGGKALDICRLYNYE---EIVIFDIPRCK-EEYINYGIIEEFKNGIVQSGKYEPVLKIVEY---VEVIVMANFLPKE-------------GIFSEDRIKVVAC          286
ABTV-[MY]_M_Rep_ABP96965                  EELMAE-PCHRRIIWVYGPNGGEGKTTYAKHLIKTRNAFYTPGGKTLDICRLYNYE---GIVIFDIPRCK-EDYLNYGILEEFKNGIIQSGKYEPVLKIVEY---VEVIVMANFLPKE-------------GIFSEDRIKLVTC          286
ABTV-[PH]_M_Rep_ABP96960                  EEIMAE-PCHRRIIWVYGPNGGEGKTTYAKHLIKTRNAFYTPGGKSLDICRLYNYE---SIVIFDIPRCK-EEYLNYGIVEEFKNGIIQSGKYEPVLKVVQY---VEVIVMANFLPKE-------------GIFSEDRIKLVTC          286
                                                                                                                                  B
```

**Figure S1. Comparison of the master Rep protein sequences of nano- and babuviruses with the Rep protein sequences of selected alphasatellites associated with nanovirids, geminiviruses and CFDV as well as two alphasatellites isolated from insects.**

Alignments were done using ClustalW in MegAlign of DNASTAR. Blue and green boxes A and B indicate the supposed M-Rep signature, i.e. two perfectly conserved sequence signatures characteristic of the M-Rep proteins of nanovirids. Names and accession numbers of the proteins are indicated on the left.

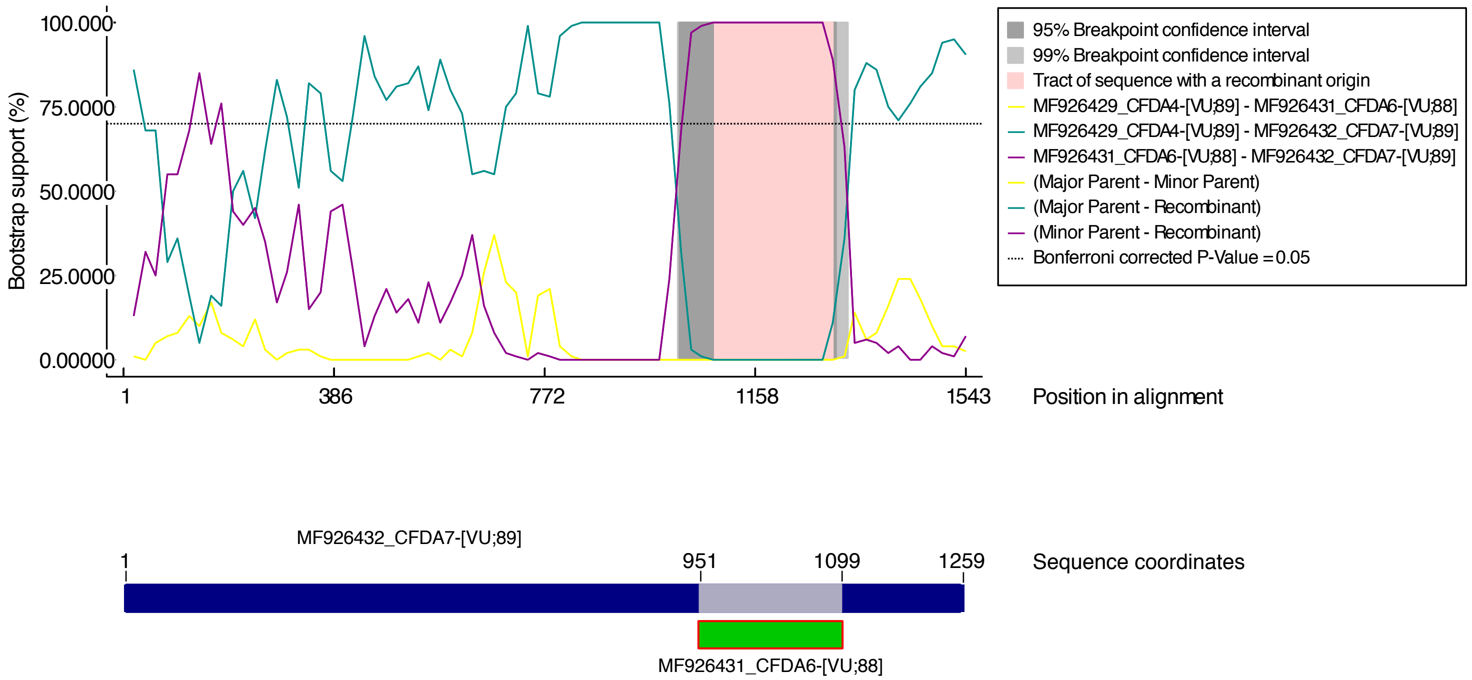


**Figure S2. Recombination analysis**

Sequences of CFD alphasatellites were aligned with sequences of ten banana bunchy top and two cardamom bushy dwarf alphasatellites using Muscle (UPGMB clustering, 8 iterations). A recombination analysis by RDP 4[26] identified CFDA7 as a potential recombinant. The recombination event was supported by RDP (*P* value 7.35 x $10^{-11}$), GENECONV (*P* 1.3 x $10^{-10}$), BootScan (*P* 1.09 x $10^{-12}$), MaxChi (*P* 2.77 x $10^{-9}$), Chimaera (*P* 3.51 x $10^{-9}$), SiScan (*P* 3.0 x $10^{-11}$) and 3Seq (*P* 1.54 x $10^{-5}$). Top panel: Bootscan plot as implemented in RDP 4 showing the recombinant region flanked by potential recombination breakpoint regions.

A graphic representation of CFDA7 with the recombinant region (position 951 to 1099) possibly derived from CFDA6 (minor parent) is shown below the plot.

Figure S3

```
                                   ATGGCTCGACAAGTTATATGXTGGTGCTTXACATTAAACAATCCTCTCTCCTCTXTCTCTTCATGAXTCAATGAAGTACCTTGTTTATCAAXTTGAACAAGGTGAATCTGGAAATATTCATTTCCAGGGTTATXTTGAAATGAAGAAX
                                            10        20        30        40        50        60        70        80        90       100       110       120       130       140       150
SCSV-[AU;F]_DNA-R_AJ290434         ATGGCTAGACAAGTTATATGCTGGTGCTTTACATTAAACAATCCTCTCGCTCCTCTCTCTCTTCATGAAAGTATGAAATACTTAGTTTATCAGACTGAAGCTGGTGATAATGGAACTATTCATTATCAAGGCTATGTGGAAATGAAGAAG
FBNYV-[EG;1-93]_DNA-R_NC_003560    ATGGCTCGGCAAGTTATATGCTGGTGCTTTACATTAAATAATCCTCTCTCTCCTCTTTCTCTTCATGATTCAATGAAGTATCTTGTTTATCAAACTGAACAAGGTGAAGCTGGAAATATTCATTTCCAGGGTTATATTGAAATGAAGAAG
MDV-[JP;1]_DNA-R_NC_003648         ATGGCTCGGCAAGTTATATGCTGGTGCTTTACATTAAATAATCCTCTCTCTCCTCTTTCTCTTCATGAATTAATGAAGTACCTTGTTTATCAAAGAGAACAAGGTGAAGCTGGAAATATTCATTTCCAGGGTTATATTGAGATGAAGAAA
FBNSV-[ET;Hol-97]_DNA-R_GU983866   ATGGCTCGGCAAGTTATATGCTGGTGCTTTACATTAAATAATCCTCTCTCTCCTATATTTCTTCATGAGTCCATGAAGTACCTTGTTTATCAAACTGAACAAGGTGAGTCTGGAAATATTCATTTCCAGGGATACATCGAAATGAAGAAA
PNYDV-[DE;15]_DNA-R_GU553134       ATGTCTCGCCAAGTTATATGTTGGTGTTTTACATTAAACAATCCTCTCTCTCTTCTCTCTCTTCATGAGTCCATGAAGTACCTTGTTTACCAACGTGAACAAGGTGAATCTGGAAATATCCATTTCCAGGGTTATGTTGAAATGAAGAAG
PYSV-[AT;15]_DNA-R_KC979054        ATGTCTCGTCAAGTTATATGCTGGTGTTTTACATTAAACAATCCTCTCTCTCCTCTCTCGCTTCATGAGTCCATGAAGTACCTTGTTTATCAACGTGAACAAGGTGAATCTGGAAATATCCATTTCCAGGGTTACATAGAAATGAAGAAG
FBYLV-[ET;231]_DNA-R_HE654123      ATGGCTCGACAAGTTATATGTTGGTGCTTTACATTAAATAATCCTCTCTCTCCTCTCTCTCTTCATGAATCAATGAAGTACCTTGTTTATCAAACTGAACAAGGTGATTCTGGAAATATTCATTTCCAGGGTTATATTGAAATGAAGAAA
BMLRV-[AT;3]_DNA-R_KC978939        ATGGCTCGTCAAGTTATATGTTGGTGCTTCACATTAAATAATCCTTCCTCCCCTCTCTCTCTTCATGAGTCCATGAAGTACCTTGTTTATCAACTAGAACAAGGTGATTCTGGAAATTTACATTTCCAGGGTTATATTGAAATGAAGAAG
BMLRV-[AZ;47]_DNA-R_KC978949       ATGGCTCGCCAAGTTATATGTTGGTGTTTCACATTAAATAATCCTTCCTCTCCTCTCTCTCTTCATGAGTCCATGAAGTACCTTGTTTATCAACAAGAACAAGGTGAATCTGGAAATATCCATTTCCAGGGTTACATAGAAATGAAGAAG
BBTV-[AU]_DNA-R_S56276             ATGGCGCGATATGTGGTATGCTGGATGTTCACCATCAACAATCCCACAACACTACCAGTGATGAGGGATGAGATAAAATATATGGTATATCAAGTGGAGAGGGGACAGGAGGGTACTCGTCATGTGCAAGGTTATGTCGAGATGAAGAGA
BBTV-[TW]_DNA-R_DQ826390           ATGGCGCGATATGTGGTATGCTGGATGTTCACCATCAACAATCCCGCTTCGCTACCAGTGATGCGGGATGAGTTCAAATATATGGTATATCAAGTGGAGAGGGGACAGGAGGGTACTCGTCATGTGCAAGGGTACGTCGAGATGAAGAGA
CBDV-[IN]_DNA-R_JX867550           ATGGCGCGATATGTGGTATGTTGGATGTTCACCATCAACAATCCCTCGTCACTACCAGTGATGAGGGATGAAATCAAATACATGGTATACCAAGTGGAGAGAGGACAGGAGGGAACCCGGCATGTGCAGGGATACGTGGAGATGAAGAGG
ABTV-[MY]_DNA-R_EF546813           ATGGCTAGATATGTCGTATGTTGGATGTTCACCATCAACAATCCCGAAGCTCTTCCAGAGATGAGGGAAGAATACAAATACCTGGTTTACCAGGTGGAGCGAGGCGAAAGCGGTACACGACATGTGCAGGGCTATGTTGAAATGAAGAGA
ABTV-[PH]_DNA-R_EF546807           ATGGCTAGGTATGTGATATGTTGGATGTTCACCATCAACAATCCCGAAACCCTTCCACCAATGAGGGATGAATACAAATACATGGTATATCAAGTAGAGCGAGGAGAAAGCGGTACTCGGCATGTGCAGGGATACGTGGAGATGAAGAGA

                                   CGAACXTCTCTXGCTGGTATGAAGAGATTXATCCCAGGTGCTCACTTTGAGAAGAGAAGAGGTACACAAGGAGAAGCTAGAGCTTAXGCTATGAAGGAAGATACAAGAATTGAAGGTCCATGGGAGTATGGTGAGTTCAAXCTCACATTT
                                           160       170       180       190       200       210       220       230       240       250       260       270       280       290       300
SCSV-[AU;F]_DNA-R_AJ290434         AGAACTTCTCTGGTGCAAATGAAGAAATTGCTTCCTGGTGCTCATCTGGAGAAGAGAAGAGGTTCACAAGGAGAAGCTAGAGCTTATGCTATGAAAGAAGATTCAAGGGTTGAAGGTCCTTGGGAGTTTGGAGAGTTTAAGGAGGTTTTG
FBNYV-[EG;1-93]_DNA-R_NC_003560    CGTACATCTCTTGCTGGTATGAAGAAATTAATTCCAGGTGCTCATTTTGAAAAGAGGAGAGGTACTCAAGGAGAAGCTAGAGCTTATTCAATGAAAGAAGATCCAAGGCTTGAAGGTCCATGGGAGTATGAAGAGTTCGTTCCCACCATT
MDV-[JP;1]_DNA-R_NC_003648         CGCACGTCTCTTGCAGGTATGAAGAAATTAATCCCAGGTGCCCACTTCGAAAAGAGGAGAGGTACTCAAGGTGAAGCTAGAGCTTACGCCATGAAGGAAGACACCCGTCTTGAAGGTCCATGGGAGTATGGGGAGTTCATCCCCACCATT
FBNSV-[ET;Hol-97]_DNA-R_GU983866   AGAACCTCCCTAGCTGGGATGAAGAGATTAATCCCAGGTGCACACTTTGAGAAGAGAAGAGGAACACAGGGAGAAGCCAGAGCTTATGCTATGAAGGAAGAATCAAGGATTGAAGGTCCATGGGAGTTTGGTGAGTTCATTGCTTCAGTT
PNYDV-[DE;15]_DNA-R_GU553134       CGTACCTCTCTTGCTGGTATGAAGAGGCTCATCCCAGGAGCTCATTTTGAGAAGAGGAGAGGTACACAAGGAGAAGCTCGTGCTTATGCTATGAAGGAAGACTCCCGTCTTGAAGGTCCATGGGAAGAAGGTGAGTTTCACCTCACAGTG
PYSV-[AT;15]_DNA-R_KC979054        CGTACGTCTTTAGCTGGTATGAAGAGGCTCATCCCAGGAGCTCACTTTGAGAAGAGGAGAGGTACTCAGGGTGAAGCTCGGGCTTATGCTATGAAGGAAAGCTCTCGTCTTGAAGGTCCATGGGAAGAAGGTGAATTCCATCTCACAGTG
FBYLV-[ET;231]_DNA-R_HE654123      CGTACGTCTCTTGCTGGTATGAAGAAACTGATACCAGGTGCTCACTTTGAGAAGAAGAAAGGCACACAAGGACAGGCCAGAGCGTATGCAATGAAAGAAGATACAAGAGTTGAAGGTCCATGGGAGTATGGTGAGTTCATTCCTACCATT
BMLRV-[AT;3]_DNA-R_KC978939        AGAACATCACTTCCTGGAATGAAGAGACTCATTCCAGGTGCTCACTTTGAAAAGAGAAGAGGTACTCAAGGAGAGGCAAGGTCATACGCCATGAAGGCCGATTCAAGAGTAGAAGGTCCTTGGGAGTATGGTGAGTTTATCTTAACCATT
BMLRV-[AZ;47]_DNA-R_KC978949       AGAACTTCTCTTGCTGCTATGAAGCGTCTAATTCCAGGTGCTCACTTTGAGAAGAGAAGAGGTACGCAAGGAGAAGCAAGAGCATACGCTATGAAGCAAGATACAAGGATGGATGGTCCTTGGGAGTATGGAGAGTTCATTCTCACCATA
BBTV-[AU]_DNA-R_S56276             CGAAGCTCTCTGAAGCAGATGAGAGGCTTCTTCCCAGGCGCACACCTTGAGAAACGAAAGGGAAGCCAAGAAGAAGCGCGGTCATACTGTATGAAGGAAGATACAAGAATCGAAGGTCCCTTCGAGTTTGGTTCATTTAAATTGTCATGT
BBTV-[TW]_DNA-R_DQ826390           CGAAGTTCTCTGAAACAGATGAGAGGCTTCTTCCCAGGCGCACACCTTGAGAAACGAAAGGGGAGCCAGGAAGAAGCACGGGCTTACTGTATGAAGGAAGATACAAGAATCGAAGGCCCCTTCGAGTTTGGTGCTTTTAAATTGTCATGT
CBDV-[IN]_DNA-R_JX867550           CGAAGCTCTCTGAAACAGATGAGGATGTTCTTCCCGGGTGCCCATCTCGAGAAGCGAAAGGGCACACAGGAAGAAGCAAGAACATACTGTATGAAGGAAGACACAAGGATCGAAGGACCCTTCGAGTTTGGGGTCTTCAAACTGGCATGT
ABTV-[MY]_DNA-R_EF546813           CGAAGTTCTCTGAAACAAATGAGGGCTTTAATTCCTGGTGCCCATCTCGAAAAGAGAAGGGGCACACAGGAAGAAGCTAGAGCTTATTGTATGAAGGCAGATACGAGAGTCGAAGGTCCCTTCGAGTTTGGTCTTTTCAAAGTATCATGT
ABTV-[PH]_DNA-R_EF546807           CGCACGTCTCTGAAACAAATGAGAGCTTTAATTCCTGGTGCCCATCTCGAGAAGAGAAAGGGCACACAGGAAGAAGCTAGATCATACTGTATGAAGGCGGATACAAGAATCGAAGGACCCTTCGAGTACGGTATGTTCAAAATATCATGT

                                   GAAGATAAGCTCAGTGATGTXATGCAGGATATGAAGAAXACXGGTAAAAGGCCTATTGAGTATATAGAAGAGTGTTGTAACACCTACGATAAATCTGCXGGTACTXTAAGXGAATTTCXAGGAGAXTTGAAGAAGAAGAAGGCTATTXXT
                                           310       320       330       340       350       360       370       380       390       400       410       420       430       440       450
SCSV-[AU;F]_DNA-R_AJ290434         GAAGATAAGCTGCGTTCTGTCATGGAAGACATGAAATCAACTGGGAAACGTCCAGTTGAGTATATAGAAGATTGTTGTAACACTTACGACAAATCTTCTGCAACGCTGAGAGAGTTTAGAGGAGAATTGAAGAAGAAACAGGCAATTGAA
FBNYV-[EG;1-93]_DNA-R_NC_003560    GAAGATAAGCTCAGAGAAGTTATGAATGATATGAAGATTACAGGTAAGAGACCTATTGAGTACATCGAAGAGTGCTGTAACACGTATGATAAATCTGCAAGTACTTTAAGAGAGTTCAGAGGTGAATTGAAGAAGAAGAAGGCTATTTCC
MDV-[JP;1]_DNA-R_NC_003648         GAAGATAAGCTCAGAGAAGTTATGAACGACATGAAGATTACAGGTAAGAGACCTATTGAGTATATAGAAGAGTGTTGTAATACATATGATAAATCTGCGAGTACTCTAAGGGAATTCAGAGGTGAGTTGAAGAAGAAGAAGGCAATTATA
FBNSV-[ET;Hol-97]_DNA-R_GU983866   GAAGATAAGCTCAGAGAAGTCATGAATGATATGAAGAACACCGGTAAGAGACCAATTGAATATATTGAAGAGTGTTGTAACACTTATGATAAGTCTGCAAGTACGCTTAGAGAATTCAGAGGAGAACTGAAGAAGAAGAAAGCTATTGCT
PNYDV-[DE;15]_DNA-R_GU553134       GAAGACAAGCTTCGTGAAGTTATGGAAGATATGAAGAACACAGGTAAACGTCCTATTGAATACATAGAAGAGTGTTGTAACACCTACGACAAGTCGTCCGGGACTCTCAGGGAATACCAAGGTGAGTTACGGAAGAAACGTGCCATTAAT
PYSV-[AT;15]_DNA-R_KC979054        GAAGATAAGCTTCGTGAAGTAATGGAAGACATGAAGAACACAGGCAAACGTCCGATTGAGTATATAGAGGAGTGCTGTAACACGTATGACAAGTCGTCTGGGACTCTCAGGGAGTATCAAGGTGAATTACGGAAGAAGCGTGCTATTTCA
FBYLV-[ET;231]_DNA-R_HE654123      GAAGATAAGCTCAGAGATGTTATGCAGGACATGAAGAACACAGGGAAGAGACCCATAGAGTATATTGAAGAGTGTTGTGATACGTATGACAAATCTGCAAGTACTCTTAGGGAATATCGAGGAGAGTTAAAGAAAAAACAAGCTATTGCA
BMLRV-[AT;3]_DNA-R_KC978939        GAAGACAAACTCAGAGAGGTTATGGGAGACATGAAGAGGACGGGTAAACGTCCTATAGAGTACATAGAAGATTGCTGTAACACATATGATAAGTCTGCTAGTACGTTGAGAGAATATAGAGGAGAGCTGAAGAAGCAGAAGGCAATTGCT
BMLRV-[AZ;47]_DNA-R_KC978949       GAAGACAAGCTCAGAGAAGTTATGGAGGACATGAAGCAGACAGGTAAGAGGCCAATTGAGTATATAGAGGATTGTTGTAACACTTACGACAAGTCAGCAAGTACTCTAAGAGAGTTTAGAGGAGAATTGAAGAAGCAGAAGGCAGTTGCT
BBTV-[AU]_DNA-R_S56276             AATGATAATTTATTTGATGTCATACAGGATATGCGTGAAACGCACAAAAGGCCTTTGGAGTATTTATATGATTGTCCTAACACCTTCGATAGAAGTAAGGATACATTATACAGAGTACAAGCAGAGATGAATAAAACGAAGGCGATGAAT
BBTV-[TW]_DNA-R_DQ826390           AATGATAATTTATTTGATGTCATACAGGATATGCGTGAAACGCATAAACGGCCTCTGGAATATTTATATGAGTGTCCGAATACCTTCGACAGAAGTAAGGATACATTATACAGAGTGCAAGCAGAGTTGAATAAAACGAAGGCGATGAAT
CBDV-[IN]_DNA-R_JX867550           AATGACAGTTTGTATGATGTAATACAGGATATGCGACTCACGAACAAAAGGCCCTTGGAGTATTTATACGATTGTCCAAATACATTCGATAGAAGTAAGGATACATTATACAGAGTACAGGCTGAGATGAATAAAACGAAAGCTATGGAG
ABTV-[MY]_DNA-R_EF546813           AATGATAATTTGTTTGATGTCATACAGGATATGAGAGAAACGCACAAACGGCCGATTGAGTATTTATACGACTGTCCTAATACCTTCGATAGAAGTAAGGATACATTATACAGGGTACAAGCGGAAATGAATAAAATGCAAGCTATGATG
ABTV-[PH]_DNA-R_EF546807           AATGATAATTTGTATGATGTCATACAGGATATGAGAGAAACGAACAAAAGGCCGATTGAGTATTTATATGATTGTCCTAATACCTTCGATAGAAGTAGAGATACATTATACAGGGTTCAGGCAGAACTCAATAAAATGCAAGCAATGAAG
```

A

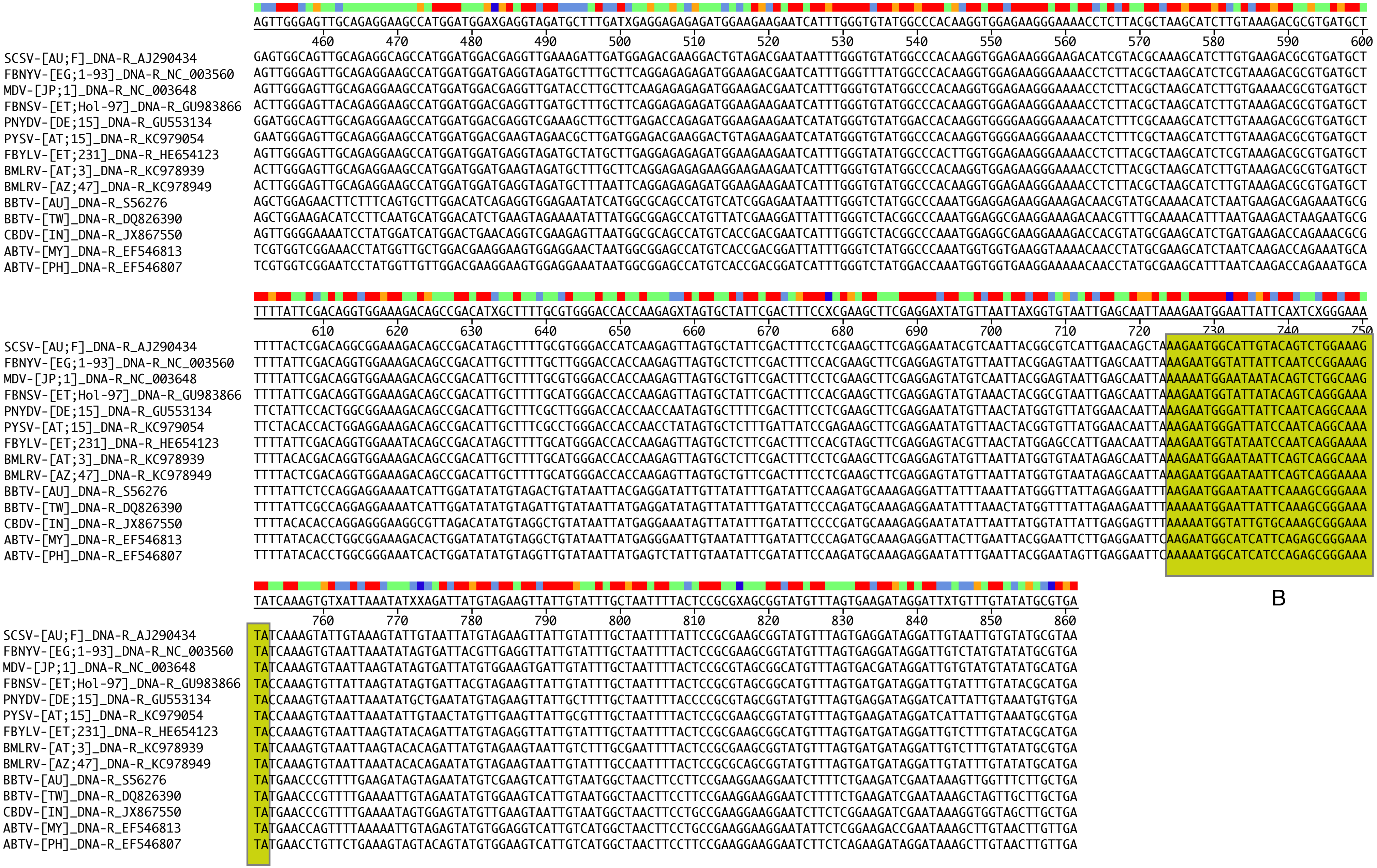

SCSV-[AU;F]_DNA-R_AJ290434
FBNYV-[EG;1-93]_DNA-R_NC_003560
MDV-[JP;1]_DNA-R_NC_003648
FBNSV-[ET;Hol-97]_DNA-R_GU983866
PNYDV-[DE;15]_DNA-R_GU553134
PYSV-[AT;15]_DNA-R_KC979054
FBYLV-[ET;231]_DNA-R_HE654123
BMLRV-[AT;3]_DNA-R_KC978939
BMLRV-[AZ;47]_DNA-R_KC978949
BBTV-[AU]_DNA-R_S56276
BBTV-[TW]_DNA-R_DQ826390
CBDV-[IN]_DNA-R_JX867550
ABTV-[MY]_DNA-R_EF546813
ABTV-[PH]_DNA-R_EF546807
B

**Figure S3. Alignment of coding sequences of nanovirus DNA-R components.**

Alignments were done using ClustalW in MegAlign of DNASTAR. Coloured boxes indicate regions corresponding to the M-Rep amino acid signature regions identified in Fig. S1, boxes A and -B, respectively. These sequences were used to design degenerate oligonucleotide primers (1 to 6, Table S3). Green boxes: fully complementary primers designed for the corresponding region, yellow and blue boxes: overlapping reverse and direct primer, respectively. Names and accession numbers of the respective nano- and babuvirus DNA-R components are indicated on the left

Figure S4

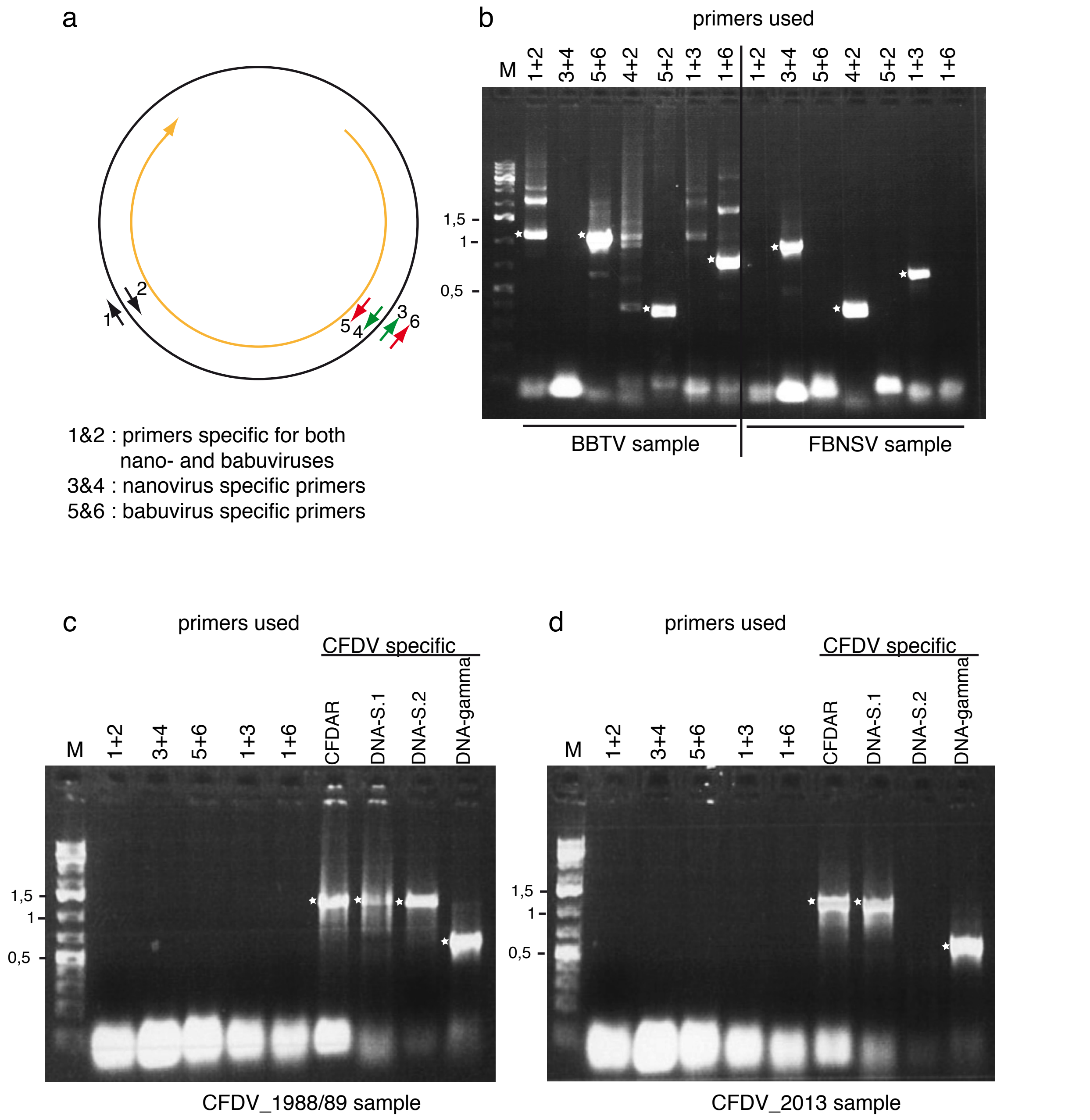


**Figure S4. PCR amplification of nanovirus DNA-R components.**
Degenerate primers (1 to 6, see Table S3) were designed based on the alignments of several babu- and nanovirus DNA-R sequences (see Fig. S3).
a - position and orientation of the primers in relation to nanovirus DNA-R.
b - control amplifications using total DNA extract of BBTV infected banana leaves (Hawaii-2013 sample, this work) or using the plasmid carrying a dimer of FBNSV DNA-R[30].
c - and d - PCR amplifications using CFDV samples. PCRs were performed on 1:10 diluted RCA products from a mixture of 23 different virion preparations of the CFDV_88/89 sample pool (c) or from a mixture of total DNA extracts from eleven different leaves of one symptomatic coconut palm for CFDV_2013 sample (d). CFDV specific primers were designed based on the cloned CFDV DNAs (the primer pairs used amplify a full-length DNA component as indicated, see Table S3).
M- 1 kb plus DNA ladder, the size (K bp) of several bands is indicated on the left of the marker, amplified DNA fragments of expected size are indicated by an asterisk.

**Table S1. CFD samples prepared from symptomatic leaves of coconut palms showing date sampled at VARTC, palm identity, virus preparation date, and characteristics of the buoyant density zone (g/ml) in a Nycodenz® isopycnic density gradient from which the sample was collected**

| Sample No (CFD) | Sampling date | Palm ID (Number of palms) | | Prep Date | Gradient density | DNA by PAGE | Virions by EM |
|---|---|---|---|---|---|---|---|
| 1 | 11.1988 | cfd2 | (1) | 28.02.89 | 1.23 | + | + |
| 2 | | cfd2 | (1) | 28.02.89 | 1.24 | + | + |
| **3**[a,b] | | cfd2 + cfd3 | (2) | 06.03.89 | 1.24 | + | + |
| 4 | | cfd2 + cfd3 | (2) | 06.03.89 | 1.24 | + | + |
| 5 | | cfd3 | (1) | 04.04.89 | 1.24 | + | n.t. |
| 6 | | cfd3 | (1) | 10.04.89 | 1.24-1.28 | + | n.t. |
| **7**[b] | 11.1988 | cfd4 | (1) | 18.04.89 | 1.23 | + | n.t. |
| 8 | 04.1989 | MRD25-21A | (1) | 13.06.89 | 1.24-1.29 | + | n.t. |
| **9**[a,b] | 09.1989 | MRD19 + MRD20 | (2) | 16.10.89 | 1.25 | + | + |
| 29 | | MRD19 + MRD20 | (2) | 16.10.89 | 1.26 | + | n.t. |
| 30 | | MRD19 + MRD20 | (2) | 16.10.89 | 1.24 | + | + |
| 31 | | MRD19 + MRD20 | (2) | 16.10.89 | 1.28 | - | - |
| 32 | | MRD19 + MRD20 | (2) | 16.10.89 | 1.23 | + | + |
| 10 | 09.1989 | MRD37.14 | (1) | 30.10.89 | 1.27 | + | n.t. |
| **11**[b] | | MRD37.14 | (1) | 30.10.89 | 1.26 | + | n.t. |
| 21[c] | 07.1988 | MRD caged | (unknown) | 16.08.88 | 1.21-1.29 | + | n.t. |
| **22**[b,c] | | MRD caged | (unknown) | 23.08.88 | 1.27-1.29 | + | n.t. |
| 23[c] | | MRD caged | (unknown) | 23.08.88 | 1.23 | + | n.t. |
| 24 | 09.1988 | TT17-7 + 12-86-14 + 12-86-15 | (3) | 07.11.88 | 1.27-1.29 | + | n.t. |
| 25 | | TT17-7 + 12-86-14 + 12-86-15 | (3) | 07.11.88 | 1.21-1.23 | + | n.t. |
| **26**[b] | | TT17-7 + 12-86-14 + 12-86-15 | (3) | 14.11.88 | 1.23-1.27 | + | + |
| 27 | | TT17-7 + 12-86-14 + 12-86-15 | (3) | 14.11.88 | 1.29 | + | + |
| 28 | | TT17-7 + 12-86-14 + 12-86-15 | (3) | 14.11.88 | 1.23 | + | + |

Samples in which DNA or virions were detected by polyacrylamide gel electrophoresis (PAGE) or electron microscopy (EM), respectively, are indicated by (+), or (-) if not detected); n.t. = not tested. MRD, Malayan Red Dwarf; cfd and other identifiers indicate that variety or hybrid source was not recorded

a – CFD samples from which RCA-DNA was used for cloning;

b – CFD samples from which RCA-DNA was pooled for deep sequencing;

c – CFD samples from caged MRD plants inoculated with viruliferous *Myndus taffini.*

**Table S2. Viral DNAs identified in two CFD samples on the basis of similar RFLP patterns and sequencing**

| Sample | DNA | Size nt | Enzyme [a] | | | | | |
|---|---|---|---|---|---|---|---|---|
| | | | *Aat*I | *Eco*RI | *Bam*HI | *Kpn*I | *Age*I | *Sal*I |
| CFD9 | DNA-S.1 | 1286 | 12 [b] | 7 | | | | |
| | DNA-S.2 | 1263 | 3 | 3 | | | | |
| | DNA-gamma | 641 | | | | 22 | | |
| | CFDAR | 1271 | | | 6 | | | |
| | CFDA2 | 1277 | | | 14 | | | |
| | CFDA3 | 1252 | | | 3 | | | |
| | CFDA4 | 1276 | | | 2 | 6 | | |
| | CFDA7 | 1259 | 3 | | | | | |
| CFD3 | CFDA1 | 1291 | | | | | 3 | |
| | CFDA6 | 1264 | | | | | | 3 |

a – DNA fragment obtained upon digestion of RCA DNA by a given enzyme was cloned into Litmus28 (for *Aat*I and *Age*I fragments) or pBluescript KSII (+)
b - number of clones showing similar RFLP pattern: insert DNAs of recombinant plasmids were amplified by PCR using M13 direct and reverse primers, and the PCR products were digested by *Hae*III or *Sau*3A enzymes.

## Table S3. Oligonucleotides used

| DNA | Primer name (direction) | Sequence (5'-3') | Tm, C° | Use [a] |
|---|---|---|---|---|
| CFDV DNA-S.1 | CFDV_S1-HindIII-dir | CCCATTAAGCTTAAAGCCCATTAAGCGATGAC | 66.9 | 1 |
| | CFDV_S1-HindIII-rev | GGCTTTAAGCTTAATGGGCCTTCACGCAATTAC | 68.2 | 1 |
| | CFDV_S1-590_dir | CATTGGCGTTTGTGCTCGATAGAG | 62.7 | 2 |
| | CFDV_S1-1160-rev | CTGCTCATCCTATGCCCGCCA | 63.7 | 2 |
| CFDV DNA-S.2 | CFDV_S2-810-BspEI-dir | TTCCGGAGATACTGCCG | 55.2 | 1, 2 |
| | CFDV_S2-810-BspEI-rev | TATCTCCGGAATACTTCA | 49.1 | 1, 2 |
| | CFDV_S2-590-dir | CATTGGCCTTGGTGTTTGGACAAAAT | 61.0 | 2 |
| | CFDV_S2-1160-rev | TCTGCTCAAGCTCTGCTCATCC | 62.1 | 2 |
| CFDV DNA-S.1 & DNA-S.2 | CFDV-CP1&2-dir | CCGGAGGAATAATGTACACGAAGAAG | 63.2 | |
| | CFDV-CP1&2-dir | CCTCCGGCATATAAAGCCGGTCTTC | 66.3 | |
| DNA-S.1 related [b] | CFDV_STL1-dir | CGGCCTAGTATTACCCGCCCACGCTC | 71.1 | 1 |
| | CFDV_STL1-rev | ACTAGGCCGCCCACGCTTATATACAGAGC | 69.5 | 1 |
| DNA-S.2 related [c] | CFDV_STL2-dir | TGCTCCCTACCTCTGCTCATCCTG | 66.1 | 1 |
| | CFDV_STL2-rev | CAGAGCAGAGGTAGTGAGC | 58.8 | 1 |
| CFDV DNA-gamma | CFDV-gamma-dir | TGGCGCTGCTCGCCCGCTTCGTCCTGGAGC | 75 | 1, 2 |
| | CFDV-gamma-rev | GCAGCGCCATTAGCTTCTTCCAATTTCACCTC | 69.5 | 1, 2 |
| CFDAR | CFDAR-BamHI-dir | CATAGAGGATCCGAAAAGAAATTTGATTCTCG | 64.4 | 1, 2 |
| | CFDAR-BamHI-rev | CTTTTCGGATCCTCTATGTACTGATACAGGAC | 66.9 | 1, 2 |
| CFDA1 | CFDA1-dir | CTTGGCTATAAATGGGTTCCTCC | 60.6 | 2 |
| | CFDA1-rev | CTTCTCCAGCAAGCTACTCACC | 62.1 | 2 |
| CFDA2 | CFDA2-392-SstI-dir | TGCTGAGCTCCGCTTGGAAGAACCTGG | 69.5 | 3 |
| | CFDA2-696-BamHI-rev | CTTCGGATCCTCTATGTACTGGTACAGTAC | 66.8 | 3 |
| | CFDA2-510-dir | ATTGGCAACTTGAGTTGCTCAG | 58.4 | 2 |
| | CFDA2-930-rev | ACAAATGCTAATTGATTATTCAAATATCAA | 55.8 | 2 |
| CFDA3 | CFDA3-398-SstI-dir | ATTTGAGCTCGGTTCCCCGGTTGTAGTTG | 68.1 | 2, 3 |
| | CFDA3-789-BamHI-rev | TTTCGGATCCTTCATCGATATAGGAAAATA | 61.3 | 2, 3 |
| CFDA4 | CFDA4-262-BamHI-dir | GACAGGATCCACCTCGAACAGGCACAC | 69.5 | 2, 3 |
| | CFDA4-563-BamHI-rev | TCGTCAGCTTGCTCTTGGATTTGAGACAGC | 68.1 | 2, 3 |
| CFDA5 | CFDA5-dir | AGCAGCCGTGCTCGACGA | 60.5 | 1, 2 |
| | CFDA5-rev | CCGATGAGCAGGGGTATG | 58.2 | 1, 2 |
| CFDA6 | CFDA6-dir | GTGGGTCCCACTTGTGGACTTTGTGGG | 69.5 | 1 |
| | CFDA6-rev | TGGGACCCACAAGGAGCGTCTGTCGAC | 71.0 | 1 |
| | CFDA6-KpnI-1159-dir | TGTGGGTACCACTTGTGGACTTTGTGGGTA | 68.1 | 1,3 |
| | CFDA6-KpnI-1172-rev | AAGTGGTACCCACAAGGAGCGTCTGTCGA | 69.5 | 1, 2,3 |
| | CFDA6-402-SstI-dir | ATTGGAGCTCGGTTCCCCGGTTCTTGTT | 68.0 | 2, 3 |
| | CFDA6-789-BamHI-rev | TTTCGGATCCTTCATCGATATAGGAAAATA | 61.3 | 3 |
| CFDA7 | CFDA7-319-SstI-dir | CATGGAGCTCCGACGCGTCCTGGTT | 69.5 | 2, 3 |
| | CFDA7-523-rev | ACCACAGCTGTAAAACCACTTTTCATTAAG | 62.7 | 2, 3 |
| CFDA8 | CFDA8-210-rev | TTGTACTGTCCGGCCACCGGAC | 65.8 | 2 |
| | CFDA8-540-dir | CTTGAATTGCTGTCTCAAATCGACGGA | 63.4 | 2 |
| | CFDA8-200-dir | AAGTCCGGTGGCCGGACAGTACAA | 66.1 | 1 |
| | CFDA8-200-rev | CCACCGGACTTCAGATGGAGGAAC | 66.1 | 1 |
| nanovirid DNA-R [d] | 1 - b-KNG-dir | AArAATGGnATnaTnCArAGCGGrAArTA | 61.7 | 1 |
| | 2 - b-KNG-rev | TAyTTyCCGCTyTGnAtnATnCCATTyTT | 61.7 | 1 |
| | 3 - nanoEYIE-rev | GAyTTrTCrTAnGTGTTACAACACTCmTCdATrTAyTC | 67.1 | 1 |
| | 4 - nanoEYIE-dir | GArTAyATHGAkGAGTGTTGTAACACnTAyGAyAArTC | 67.1 | 1 |
| | 5 - babu-EYL-dir | AATACCTTCGAyAGAAGTArAGATACATTATACAG | 63.6 | 1 |
| | 6 - babuEYL-rev | rTCGAAGGTATThGGACArTCrTATAAATACTC | 64.3 | 1 |

a – a pair of primers indicated by the same number was used for PCR amplification of the corresponding DNA for the following purposes: (1) full-length cloning, (2) component-specific detection by PCR, (3) amplification of the fragment for construction of redundant copies of the component;
b – primers designed based on the DNA sequence in the region with potential stem-loop structure (STL) of CFDV DNA-S.1;
c – primers designed on the basis of the DNA sequence of STL region of CFDV_DNA-S.2;
d – primers designed on the basis of the alignment of the nanovirid DNA-R sequences.

**Table S4. Mapping of primary reads from deep sequencing data of three BBTV samples**

| Sample / DNA | BBTV-Hawaii_2013 (26,758,442 total reads) | | | BBTV-Nigeria_2013 (25,761,084 total reads) | | | BBTV-Vietnam_2013 (34,536,988 total reads) | | |
|---|---|---|---|---|---|---|---|---|---|
| | Reads | % of reads | Cov. Mean | Reads | % of reads | Cov. Mean | Reads | % of reads | Cov. Mean |
| DNA-R | 1,671,973 | 6.3 | 149,484 | 562,089 | 2.2 | 50,015 | 731,405 | 2.1 | 65,039 |
| DNA-S | 571,790 | 2.1 | 50,314 | 67,470 | 0.3 | 5,952 | 3,042,114 | 8.8 | 271,294 |
| DNA-M | 1,306,112 | 4.9 | 122,536 | 264,262 | 1 | 24,898 | 1,302,658 | 3.8 | 124,545 |
| DNA-N | 7,240,846 | 27.1 | 657,758 | 6,323,995 | 24.6 | 565,927 | 5,100,799 | 14.8 | 460,561 |
| DNA-C | 5,137,818 | 19.2 | 502,970 | 893,580 | 3.5 | 87,631 | 850,602 | 2.5 | 82,171 |
| DNA-U3 | 8,579,130 | 32.1 | 797,873 | 5,282,003 | 20.5 | 487,589 | 3,922,111 | 11.4 | 363,683 |
| BBTA2[a] | | | | | | | 3,161,344 | 9.2 | 279,229 |
| total | 24,507,669 | 91.6 | | 13,393,399 | 52 | | 18,111,033 | 52.4 | |

Geneious mapping of total reads against all identified (*de novo* assembled) BBTV DNAs, performed with the settings allowing 10% mismatch per read. Cov. Mean – average coverage.
a - a variant of the proposed alphasatellite species BBTA2[25]

**Table S5. CFDV mutant spectra**

| DNA | Year | Type of base changes | | | | | | | | | | | | | | | | |
|---|---|---|---|---|---|---|---|---|---|---|---|---|---|---|---|---|---|---|
| | | Total | A C | A G | A T | C A | C G | C T | G A | G C | G T | T A | T C | T G | transitions | transversions | deletions | insertions |
| DNA-R | 1989 | | | | | | | | | | | | | | 0 | 0 | | |
| | 2013 | 2 | | | | | | | | 1 | | | | | 0 | 1 | | 1 (+1) |
| DNA-S.1 | 1989 | | | | | | | | | | | | | | | | | |
| | 2013 | 1 | | | | | | | | | | | | 1 | | | | |
| | 2015 | 0 | | | | | | | | | | | | | 0 | 0 | | |
| DNA-S.2 | 1989 | | | | | | | 1 | | | | | 1 | 1 | 2 | 1 | | |
| | 2015 | 3 | | | | | | | | | | | | | 0 | 0 | | |
| CFDA1 | 1985 | | | | | | | | | | | | | | 0 | 0 | | |
| | 1988 | 2 | | | | | | 1 | 1 | | | | | | 2 | 0 | | |
| | 1989 | 1 | | | | | | | | | | | 1 | | 1 | 0 | | |
| | 2013 | 20 | 1 | 1 | | | 2 | 3 | 3 | 2 | 3 | | 1 | 2 | 7 | 11 | 1 (-3) | 1 (+1) |

Number and type of changes between the most recent and respective previous sampling dates are shown. For calculation of the nucleotide substitution rates per site per year the deletion of three nucleotides is counted as one event.

**Table S6. Cloning of redundant copies of CFD associated DNAs used for replication assays**

| DNA | Primers used for PCR amplification of monomer [a] | Monomer [b] | Primers used for PCR amplification of 0,2 mer [c] | Redundant copy in pBin19 |
|---|---|---|---|---|
| CFDV DNA-S.1 | CFDV_S1-HindIII-dir<br>CFDV_S1-HindIII-rev | pBluescriptIIKS(+)-HindIII-S1-5 | | Dimer first obtained by cloning a monomer into pBluescriptIIKS(+) HindIII site and then transferred into pBin19 as BamHI-KpnI fragment |
| CFDV DNA-S.2 | CFDV_S2-810-BspEI-dir<br>CFDV_S2-810-BspEI-rev | pBluescriptIIKS(+)-BspEI-S2-4 | | Dimer first obtained by cloning a monomer into pBluescriptIIKS(+) BspEI site and then transferred into pBin19 as BamHI-KpnI fragment |
| CFDV DNA-gamma | | pBluescriptIIKS(+)-KpnI-mini-6 | | Direct cloning of a monomer into KpnI site of pBin19 with selection of dimer insertion |
| CFDAR | CFDAR-BamHI-dir<br>CFDAR-BamHI-rev | pBluescriptIIKS(+)-BamHI-AR-1 | | Direct cloning of a monomer into BamHI site of pBin19 with selection of dimer insertion |
| CFDA1 | | Litmus28-AgeI-A1-6 [d] | | Dimer first obtained by cloning a monomer into Litmus28 AgeI site and then transferred into pBin19 as BamHI-KpnI fragment |
| CFDA2 | | pBluescriptIIKS(+)-BamHI-A2-2 | CFDA2-392-SstI-dir<br>CFDA2-696-BamHI-rev | 302 bp fragment was PCR amplified using indicated primers, digested by SstI and inserted into pBin19-(SstI-HindIIIblunt) [e] plasmid. CFDA2 monomer was consequently inserted into BamHI site of this plasmid thus giving rise to CFDA2 1,2 mer. |
| CFDA3 | | pBluescriptIIKS(+)-BamHI-A3-24 | CFDA3-398-SstI-dir<br>CFDA3-789-BamHI-rev | 390 bp fragment was PCR amplified using indicated primers, digested by SstI and inserted into pBin19-(SstI-HindIIIblunt) plasmid. CFDA3 monomer was subsequently inserted into BamHI site of this plasmid thus giving rise to CFDA3 1,2 mer. |
| CFDA4 | | pBluescriptIIKS(+)-BamHI-A4-14 | CFDA4-262-BamHI-dir<br>CFDA4-563-BamHI-rev | 300 bp fragment was PCR amplified using indicated primers, digested by BamHI and inserted into pBin19-(BamHI-HindIIIblunt) [f] plasmid. CFDA4 monomer was consequently inserted into BamHI site of this plasmid thus giving rise to CFDA2 1,2 mer. |
| CFDA6 | CFDA6-KpnI-1159-dir<br>CFDA6-KpnI-1172-rev | pBluescriptIIKS(+)-HincII-A6-7 | CFDA6-402-SstI-dir<br>CFDA6-789-BamHI-rev | 360 bp fragment was PCR amplified using indicated primers, digested by SstI and inserted into pBin19-(SstI-HindIIIblunt) plasmid. CFDA6 monomer, liberated as KpnI fragment, was ligated, ligation product digested by BamHI and inserted into BamHII site of the plasmid with cloned 360 fragment, thus giving rise to CFDA6 1,2 mer. |

| | | | |
|---|---|---|---|
| CFDA7 | Litmus28-AatII-A7-15 | CFDA7-319-SstI-dir<br>CFDA7-523-rev | 320 bp fragment was PCR amplified using indicated primers, digested by SstI and inserted into pBin19-(SstI-HindIIIblunt) plasmid. CFDA7 monomer, liberated as AatI fragment, was ligated, ligation product digested by HindIII and inserted into HindIII site of the plasmid with cloned 320 fragment, thus giving rise to CFDA7 1,2 mer |

a - PCR performed on RCA DNA of sample CFD_88/89

b - plasmids used to clone a given DNA in the restriction site indicated, numbers at the end identify the recombinant plasmids

c - viral DNA from the plasmid shown under "Monomer" was used for PCR amplification

d - if primers are not indicated in the second column, viral DNA was cloned as monomer after restriction of RCA DNA into the corresponding plasmid, see Table S2 for enzymes used.

e - pBluescriptIIKS(+) plasmid was digested by *Hind*III, the ssDNA ends resulting after digestion were filled using dNTPs and Klenow polymerase. After purification by phenol extraction and ethanol precipitation the plasmid DNA was digested by *Sst*I.

f - pBluescriptIIKS(+) plasmid was digested by *Hind*III, the ssDNA ends resulting after digestion were filled using dNTPs and Klenow polymerase. After purification by phenol extraction and ethanol precipitation the plasmid DNA was digested by *Bam*HI.

**Table S7. GenBank accession numbers of CFD associated DNAs**

| DNA \ Year | 1985 | 1988 | 1989 | 2013 | 2015 |
|---|---|---|---|---|---|
| DNA-S.1 | | | MF926436 | MF926437 | MF926438 |
| DNA-S.2 | | | MF926439 | | MF926440 |
| DNA-gamma | | | MF926441 | MF926442 | MF926443 |
| CFDAR | | | MF926434 | MF926435 | |
| CFDA1 | M29963 | MF926423 | MF926424 | MF926425 | |
| CFDA2 | | | MF926426 | | |
| CFDA3 | | | MF926427 | | |
| CFDA4 | | | MF926429 | MF926444 | |
| CFDA5 | | | MF926430 | | |
| CFDA6 | | MF926431 | | | |
| CFDA7 | | | MF926432 | | |
| CFDA8 | | | | | MF926433 |
